# PIMT regulates hepatic gluconeogenesis in mice

**DOI:** 10.1016/j.isci.2023.106120

**Published:** 2023-02-02

**Authors:** Bandish Kapadia, Soma Behera, Sireesh T. Kumar, Tapan Shah, Rebecca Kristina Edwin, Phanithi Prakash Babu, Partha Chakrabarti, Kishore V.L. Parsa, Parimal Misra

**Affiliations:** 1 Center for Innovation in Molecular and Pharmaceutical Sciences, Dr. Reddy’s Institute of Life Sciences (DRILS), University of Hyderabad Campus, Hyderabad, TG 500046, India; 2 Department of Biotechnology, University of Hyderabad, Hyderabad 500046, India; 3 Department of Biochemistry, Saurashtra University, Rajkot 360005, India; 4 Indian Institute of Chemical Biology, Jadavpur, Kolkata 700032, India

**Keywords:** Hepatology, Human metabolism, Molecular biology

## Abstract

The physiological and metabolic functions of PIMT/TGS1, a third-generation transcriptional apparatus protein, in glucose homeostasis sustenance are unclear. Here, we observed that the expression of PIMT was upregulated in the livers of short-term fasted and obese mice. Lentiviruses expressing Tgs1-specific shRNA or cDNA were injected into wild-type mice. Gene expression, hepatic glucose output, glucose tolerance, and insulin sensitivity were evaluated in mice and primary hepatocytes. Genetic modulation of PIMT exerted a direct positive impact on the gluconeogenic gene expression program and hepatic glucose output. Molecular studies utilizing cultured cells, *in vivo* models, genetic manipulation, and PKA pharmacological inhibition establish that PKA regulates PIMT at post-transcriptional/translational and post-translational levels. PKA enhanced 3′UTR-mediated translation of TGS1 mRNA and phosphorylated PIMT at Ser656, increasing Ep300-mediated gluconeogenic transcriptional activity. The PKA-PIMT-Ep300 signaling module and associated PIMT regulation may serve as a key driver of gluconeogenesis, positioning PIMT as a critical hepatic glucose sensor.

## Highlights

• PIMT protein levels are enhanced upon fasting
• Hepatic expression of PIMT is increased in HFD and obese mice
• PIMT is a PKA substrate and phosphorylation of PIMT augments its gluconeogenic ability
• Depletion of PIMT improves glucose clearance capacity and enhances insulin sensitivity

## Graphical abstract

**Figure:**
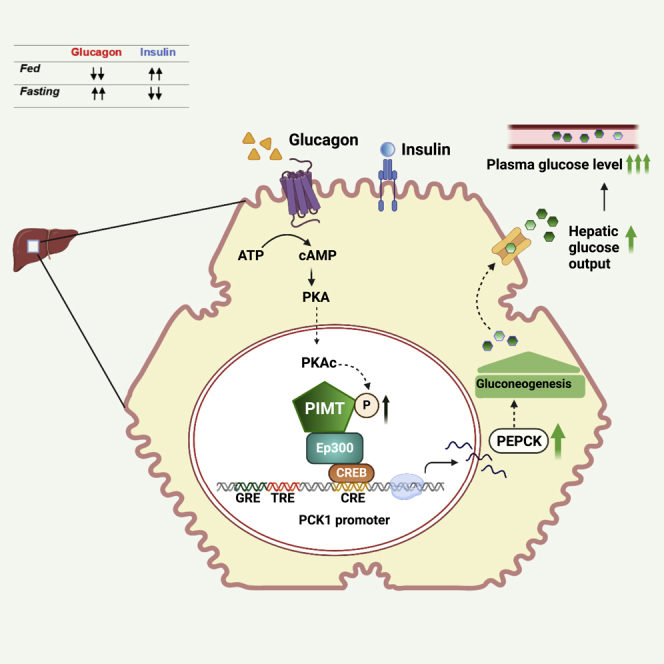

## Introduction

In mammals, the transition between fasting and fed state is accompanied by complex hepatic gene expression changes. The liver is the central hub for coordinating the fasting-feeding transitions, given its role in maintaining blood glucose levels via processing the dietary intake and maintaining whole-body nutritional/energy balance. Several extracellular factors tightly coordinated this hepatic switch on/off mechanism, thereby controlling insulin and glucagon levels. Elucidating the complex metabolic changes associated with fasting and feeding and their transcriptional underpinnings is crucial for understanding physiology and metabolic dysfunctions such as insulin resistance.

During fasting, evolutionarily conserved glucagon stimulates GPCR-induced cAMP-activated PKA-driven transcriptional signatures in hepatocytes, augments the expression of critical gluconeogenic regulatory genes, including phosphoenolpyruvate carboxykinase 1 *(PCK1)* and glucose-6-phosphatase catalytic subunit 1 *(G6PC1)*. This promoter-regulated rate-limiting gluconeogenic enzyme expression is vital for homeostasis and provides an efficient adaptive mechanism to the external clues.^1^^,^^2^^,^^3^ Several factors, such as a high energy-rich diet, disrupt glucose-sensitive metabolic switches leading to metabolic alterations and the development of T2D. In eukaryotes, numerous nuclear receptors and transcription factors, stimulated by different hormones, regulate the gene expression to sustain hepatic glucose production.^1^^,^^3^ The lack of a direct association between specific transcription factors and upstream regulatory nodes led to the discovery of second-generation transcriptional regulators, the co-regulators. By converging signals to the transcription factors/nuclear receptors via a multi-leveled signaling cascade, co-regulators function as essential signaling integrators for coordinating broad gluconeogenic transcriptional programs.^4^^,^^5^ Such multi-protein regulatory complexes fine-tune gluconeogenic gene expression patterns and thus confer the second level of specificity in the transcriptional regulation,^6^^,^^7^ an event typically hijacked in metabolic syndrome. Although significant progress has been made in understanding the complex relationships between hormonal signaling, whole-body metabolism, and liver machinery for glucose production, the lack of a unique convergence signaling node guiding the multi-protein co-regulator complex for integrating the diverse extracellular signaling cues remains to be elucidated.

PIMT/TGS1 (PRIP Interacting protein with Methyl Transferase domain/Trimethyl guanosine synthase I) was first reported from Prof. Janardan Reddy’s lab using PRIP (peroxisome proliferators-activated receptor (PPARγ)-interacting protein) as a bait in a yeast two-hybrid study.^8^ A detailed *in vitro* proteome interaction of the protein revealed that PIMT interacts with several co-activators encompassing HAT complex and mediator complex proteins. This comprehensive report revealed differential modulation of co-activator-driven transcriptional complexes by PIMT, proposing the protein acts as a key node in switching the HAT complex with the Mediator complex for enhanced mRNA synthesis.^9^ Besides the transcriptional regulation, protein also participates in evolutionarily conserved hypermethylation of small non-coding RNA. Studies from Drosophila^10^ and mice models establish that PIMT is essential for development.^11^

Previous work from our lab established that PIMT is an essential component of nuclear receptor-driven transcriptional regulations.^12^^,^^13^ Furthermore, kinase-dependent phosphorylation of PIMT in the recruitment of transcriptional co-regulators has positioned PIMT as a critical player in hormonal signaling.^13^ Indeed, inflammation-induced PIMT expression in skeletal muscle hampers insulin signaling leading to insulin resistance via the transcriptional downregulation of MEF2A and GLUT4 and attenuation of Akt phosphorylation.^12^ Our previous studies also showed that ERK/hyperthyroidism-induced phosphorylation of PIMT at Ser^298^ was required for enhanced gluconeogenesis, suggesting that PIMT may be a driver of pre-insulin resistance conditions.^13^ Recently, PIMT was reported to be a key player regulating β-cell mass and function.^14^ Although PIMT was known to play an essential role under pathological relevant conditions, the importance of PIMT in controlling glucose homeostasis during short-term physiological fasting and hepatic insulin resistance conditions remained elusive. In the current study, we observed that the expression of PIMT was upregulated in a post-transcriptional/translational-dependent manner upon short-term fasting. Furthermore, glucagon-induced PKA regulated PIMT activity/levels at the gene expression level along with its transcriptional activity. Notably, depletion of PIMT in pathological relevant insulin resistance animal models displayed significant improvement in hepatic insulin sensitivity positioning PIMT as a druggable target.

## Results

### Fasting responsive signaling pathway regulates hepatic PIMT expression

**Figure 1:**
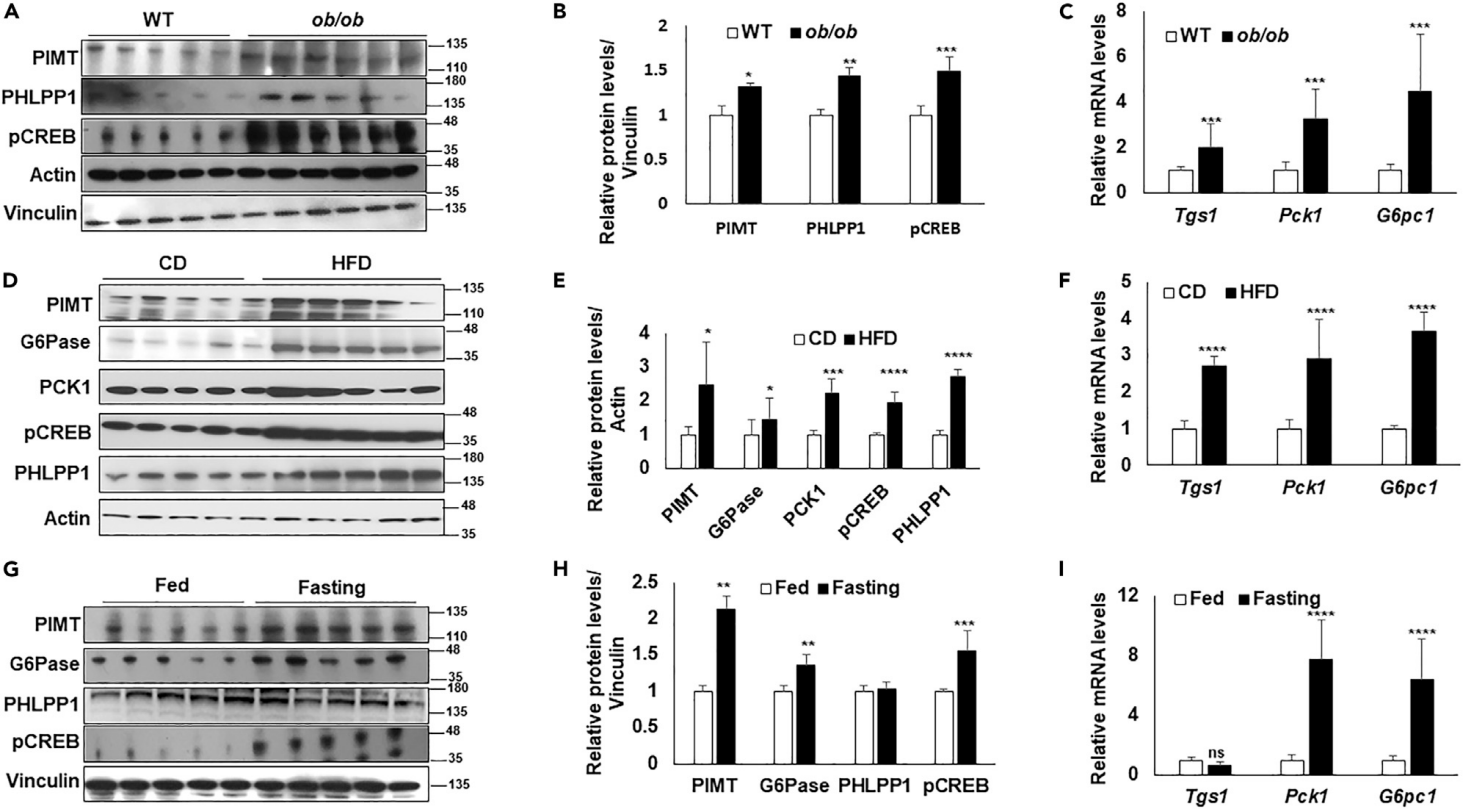
Hepatic expression of PIMT is upregulated upon nutritional stress (A) Immunoblot analysis indicated antibodies in the liver lysate of wild-type (WT) or *ob/ob* mice (n = 5). (B) Quantitative densitometry evaluation of Figure 1A. Values were normalized with the corresponding loading control, Vinculin. (C) qPCR analysis of the indicated genes in the liver lysate of WT and *ob/ob* mice (n = 5). Values were normalized using 18S as a reference gene (n = 5). (D) Immunoblot analysis indicated antibodies in the liver lysate of mice fed on a chow diet (CD, n = 5) or high-fat diet (HFD, n = 6). (E) Quantitative densitometry evaluation of Figure 1D. Values were normalized with the corresponding loading control, actin. (F) qPCR analysis of the indicated genes in the liver lysate of CD (n = 5) and HFD mice (n = 6). Values were normalized using 18S as a reference gene. (G) Immunoblot analysis indicated antibodies in the liver lysate of wild-type fed, or 8h fasted mice (n = 5). (H) Quantitative densitometry evaluation of Figure 1G. Values were normalized with the corresponding loading control, Vinculin. (I) qPCR analysis of the indicated genes in the liver lysate of wild-type fed or 8h fasted mice (n = 5). Values were normalized using 18S as a reference gene. Statistical analysis was performed using unpaired Student’s *t* test (two-tailed) ^∗^*p<*0.05, ^∗∗^*p<*0.01, ^∗∗∗^*p<*0.005, *∗∗∗∗p<*0.001 versus the corresponding controls.

To gain a deeper understanding of the role of PIMT in glucose homeostasis, we investigated the hepatic expression of PIMT in obese and short-term fasted mice. We observed that the levels of PIMT protein were increased in the livers of two obesity-associated T2D mouse models: *ob/ob* mice (Figures 1A and 1B) and wild-type (C57BL/6) mice fed with the high-fat diet (HFD) (Figures 1D and 1E) compared with the wild-type and control diet-fed mice, respectively. Consistent with our previous report,^12^ the mRNA level of *Tgs1* was enhanced in the diabetic mice models (Figures 1C and 1F). Importantly, the phosphorylation of CREB, an established downstream mediator of the Glucagon-cAMP-PKA axis,^15^ was found to be enhanced (Figures 1A and 1D). Furthermore, protein levels of PHLPP1, a Ser/Thr phosphatase, earlier reported by us to inhibit insulin signaling cascade in the skeletal muscle, were also modestly increased in the livers of *ob*/*ob* and HFD-fed mice (Figures 1A, 1B, 1D, and 1E).^16^ PIMT protein abundance and the levels of fasting-sensitive pCREB and G6Pase were increased in mice subjected to 8h fasting (short-term fasting) (Figures 1G and 1H), however, PHLPP1 levels remained largely unchanged. While the transcript levels of *Pck1* and *G6pc1* were elevated in short-term fasted mice as anticipated, the mRNA level of *Tgs1* was unaltered (Figure 1I). To further study the impact of nutritional status and hormonal dependency on PIMT expression, mice were subjected to fasting followed by re-feeding. As expected, 8h fasting induced the expression of *Pck1* and *G6pc1* (Figure S1) but not PHLPP1. Consistent with our previous observations, PIMT protein levels, but not mRNA levels, were enhanced upon fasting and were normalized upon 8 h of re-feeding (Figures S1A–S1C). Thus, the induction of PIMT is a relatively early event suggesting that glucagon-cAMP-PKA signaling regulates its hepatic level.

### Hepatic PKA signaling augments PIMT protein levels

**Figure 2:**
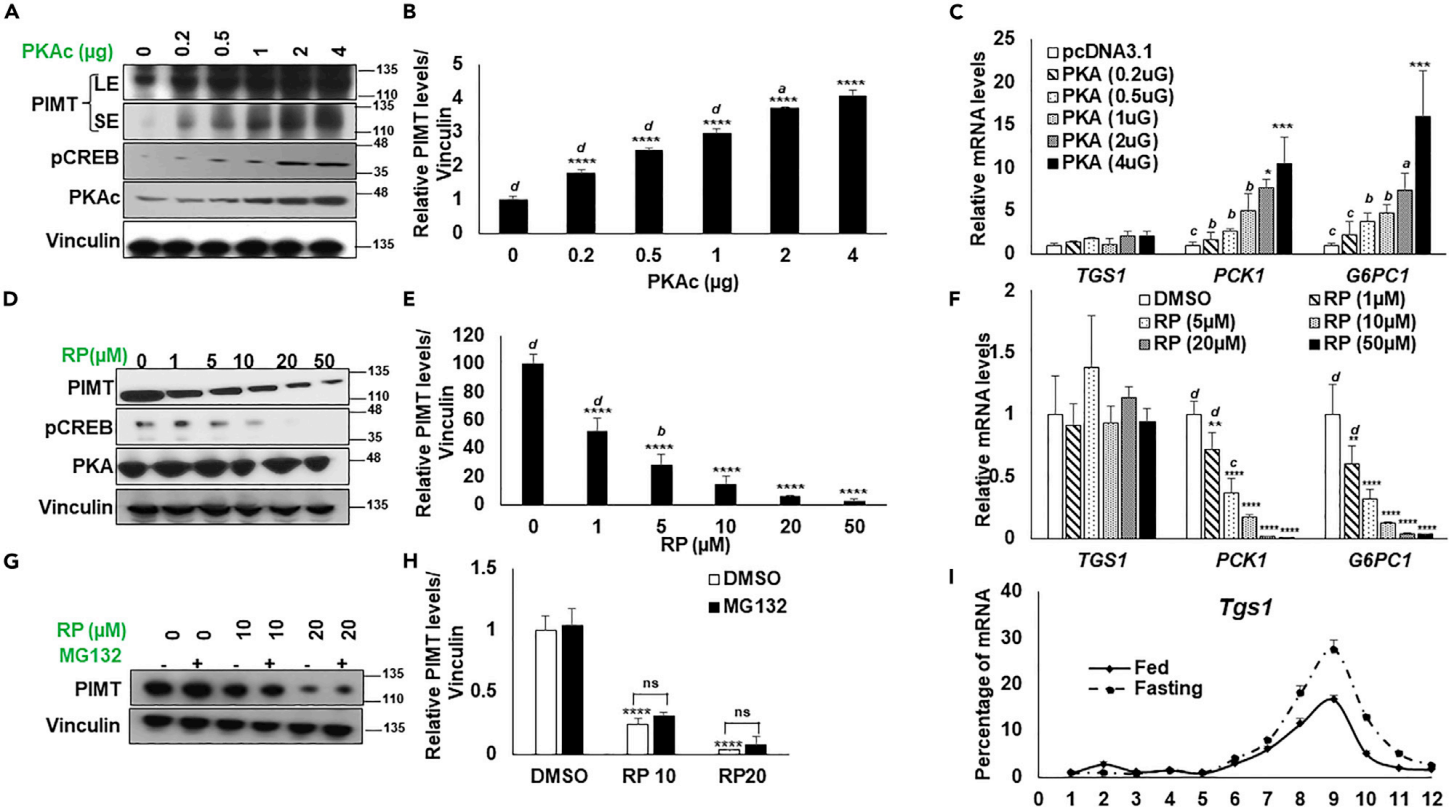
Hepatic PKA regulates PIMT protein expression (A) HepG2 cells were transfected with an increasing concentration of active catalytic subunit of PKA (PKAc). Post 48h of transfection, cells were lysed, and immunoblots assessed the protein levels of PIMT. (B) Densitometry quantification of Figure 2A. Values were normalized with the corresponding loading control, Vinculin. Data are representative of 3 independent experiments and are expressed as mean ± SD. Statistical analysis was performed using one-way ANOVA followed by Bonferroni’s post hoc test. ^∗∗∗∗^*p<* 0.001 compared to pcDNA3.1 transfected cells, ^*a*^p<0.05, ^*d*^p<0.001 vs PKAc (4 μg) transfected cells. (C) qPCR analysis of the indicated genes upon PKAc overexpression in HepG2 cells. Values were normalized using 18S as a reference gene. Data are representative of 3 independent experiments and are expressed as mean ± SD. Statistical analysis was performed using one-way ANOVA followed by Bonferroni’s post hoc test. ^∗∗^*p<*0.01, ^∗∗∗^*p<*0.005, ^∗∗∗∗^*p<*0.001 compared to pcDNA3.1 transfected cells, ^*a*^*p*<0.05, ^*b*^*p*<0.01, ^*d*^*p*<0.001 vs PKAc (4 μg) transfected cells. (D) HepG2 cells were treated with increasing concentrations of Rp-Br-cAMPs (RP). Post 8h of treatment, cells were lysed, and immunoblots assessed the protein levels of PIMT. (E) Densitometry quantification of Figure 2D. Values were normalized with the corresponding loading control, Vinculin. Data are representative of 3 independent experiments and are expressed as mean ± SD. Statistical analysis was performed using one-way ANOVA followed by Bonferroni’s post hoc test. ^∗∗∗∗^p*<*0.001 compared to DMSO treated cells, ^*b*^*p*<0.01, ^*d*^*p*<0.001 vs RP (50 μM) treated cells. (F) qPCR analysis of the indicated genes upon RP treatment in HepG2 cells for 8h. Values were normalized using 18S as a reference gene. Data are representative of 3 independent experiments and are expressed as mean ± SD. Statistical analysis was performed using one-way ANOVA followed by Dunnett’s post hoc test. ^∗∗^*p<*0.01, ^∗∗∗^*p<*0.005, ^∗∗∗∗^*p<*0.001 compared to DMSO treated cells, ^*c*^*p*<0.005, ^*d*^*p*<0.001 vs RP (50 μM) treated cells. (G) HepG2 cells were treated with increasing concentration of RP for 4h followed by treatment with MG132 for 4h (10 μM). Post-treatment cells were lysed and probed for endogenous PIMT protein levels. (H) Densitometry quantification of Figure 2G. Values were normalized with the corresponding loading control, Vinculin. Data are representative of 3 independent experiments and are expressed as mean ± SD. Statistical analysis was performed using one-way ANOVA followed by Bonferroni’s post hoc test. ^∗∗∗∗^*p<*0.001 compared to DMSO treated cells, ns: non-significant. (I) qPCR of the sucrose gradient fractions from fed and fasting liver lysates of mice. The values were normalized with extracellular Luciferase RNA (n = 4).

Alterations in the PIMT protein levels during fasting prompted us to examine whether glucagon-cAMP-induced PKA signaling impacts PIMT protein levels. For this, the catalytic alpha subunit of the PKA holoenzyme complex (PKAc) was overexpressed in HepG2 cells in a dose-dependent manner, and levels of PIMT protein were examined. A dose-dependent increase of PKAc enhanced pCREB levels (Figure 2A). More importantly, PIMT protein levels, but not mRNA levels, were enhanced in a PKA-dose-dependent fashion (Figures 2A–2C). mRNA levels of gluconeogenic genes, *PCK1*, and *G6PC1* were also augmented in a dose-dependent manner (Figure 2C). Supporting our findings, PIMT protein level but not its transcript levels were increased in HepG2 cells treated with increasing concentrations of Forskolin, an adenyl cyclase activator (Figures S2A–S2C). Finally, corroborating the findings that intact PKA activity is responsible for PIMT protein induction, we treated the cells with two independent PKA inhibitors, H89 and Rp-8-Br-cAMPs (RP), and examined PIMT protein levels by immunoblotting. We noted that the inhibition of PKA activity significantly depleted endogenous PIMT protein but not mRNA levels (Figures 2D–2F and S2D–S2F). Transcript levels of *PCK1* and *G6PC1* served as the internal controls (Figures 2F and S2F). Taken together, these results indicate that fasting-induced PKA signaling is required to induce PIMT protein levels during fasting.

We next investigated the molecular events underlying the PKA-regulated PIMT expression. The increase in the protein levels of PIMT was not associated with elevated mRNA levels, suggesting that the alteration was likely independent of its transcriptional regulation. Therefore, we explored whether PIMT protein stability is affected by PKA signaling. Thus, we overexpressed SFB-PIMT in HepG2 cells and altered PKA activity to study its impact on the overall protein levels. Surprisingly, treatment with two independent PKA inhibitors (H89 and RP) or PKA activator Forskolin (FSK) showed minimal impact on overall flag antibody signals (Figure S3A). Next, we treated HepG2 cells with a proteasomal degradation complex inhibitor, MG132. We observed that the PIMT protein levels were not impacted (Figures 2G and 2H), indicating that the PKA signaling does not alter PIMT protein stability. Next, we questioned whether PKA signaling affects the post-transcriptional regulation of PIMT. Here, we transfected HepG2 cells with a luciferase reporter construct fused with the 3′UTR of *TGS1* at its 3′ end, followed by treatment with PKA modulators. Post-treatment, cells were lysed, and luciferase activity was measured. Treatment with PKA inhibitors significantly reduced the luciferase readout, while exposure to Forskolin enhanced the luciferase expression (Figure S3B). To ascertain that the modulation of PIMT expression is regulated at 3′UTR in a PKA-dependent manner, we co-expressed PKAc and luciferase reporter constructs in HepG2 cells. We observed that the activity was upregulated in a dose-dependent manner (Figure S3C). Empty Luciferase reporter vector was used as the internal control. To examine further, we performed sucrose gradient density fractionation of the livers of fasted mice. Consistently, *Tgs1* mRNA levels were observed to be enriched in actively translating ribosomal fractions (Figure 2I), suggesting that PKA signaling may potentiate PIMT levels by enhancing the translation of T*gs1*.

### PIMT regulated gluconeogenesis *in vivo* and *in vitro*

**Figure 3:**
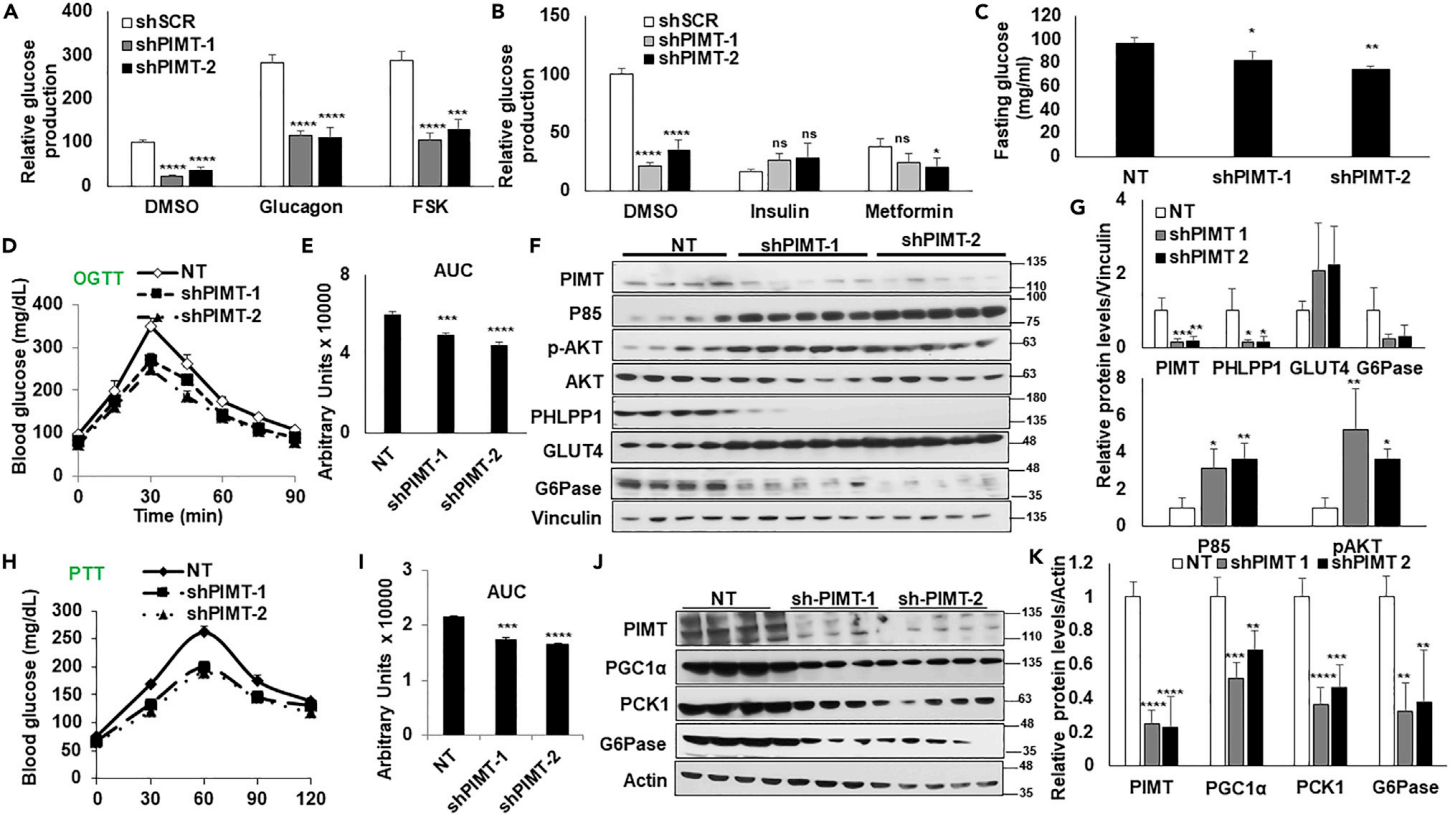
Hepatic PIMT expression is essential for fasting-inducing glucose production (A and B) Primary hepatocytes isolated from three different female mice were infected with lentiviruses expressing shRNA against *Tgs1* (two independent shRNA). shSCR (nonspecific control) was used as the internal control. Post 48h of infections, cells were cultured in glucose production media and indicated treatments, followed by an estimation of glucose released in the media. Data are representative of 3 independent experiments and are expressed as mean ± SD. Statistical analysis was performed using one-way ANOVA followed by Dunnett’s post hoc test. ^∗^*p<*0.05, *∗∗∗∗p<*0.001 compared to control infected cells. (C) C57BL/6 male mice were tail-vein injected with lentivirus expressing shRNA against *Tgs1* (two independent shRNA) (n = 5). shSCR tail-vein injections were used as the internal control. Post 7 days of injection, mice fasted for 8h. Post-fasting, glucose was quantified from tail vein. Numerical data are expressed as mean ± SD. Statistical analysis was performed using one-way ANOVA followed by Dunnett’s post hoc test. ^∗^*p<*0.05, ^∗∗^*p<*0.01 compared to control fasting mice. (D) Oral Glucose tolerance test in C57BL/6J male mice expressing shRNA against *Tgs1* (two independent shRNA) in the liver (n = 4). (E) The area under the curve for Figure 3D. Numerical data are expressed as mean ± SD. Statistical analysis was performed using one-way ANOVA followed by Dunnett’s post hoc test. ^∗∗∗^*p<*0.005, ^∗∗∗∗^*p<*0.001 compared to NT-infected mice. (F) Immunoblot analysis of the liver lysates using the indicated antibodies. (G) Densitometric quantification of Figure 3F. Values were normalized with the corresponding loading control, Vinculin. Numerical data are expressed as mean ± SD. Statistical analysis was performed using one-way ANOVA followed by Dunnett’s post hoc test. ∗p *<* 0.05, ^∗∗^*p<*0.01, ^∗∗∗^*p<*0.005 compared to NT-infected mice. (H) Pyruvate tolerance test in C57BL/6J mice expressing shRNA against *Tgs1* (two independent shRNA) in the liver (n = 4). (I) The area under the curve for Figure 3H. Numerical data are expressed as mean ± SD. Statistical analysis was performed using one-way ANOVA followed by Dunnett’s post hoc test. ^∗∗*∗*^*p<*0.005, ^∗∗∗∗^*p<*0.001 compared to NT-infected mice. (J) Immunoblot analysis of the liver lysates using the indicated antibodies. (K) Densitometric quantification of Figure 3J. Values were normalized with the corresponding loading control, actin. Numerical data are expressed as mean ± SD. Statistical analysis was performed using one-way ANOVA followed by Dunnett’s post hoc test. ^∗∗^*p<*0.01, ^∗∗∗^*p<*0.005, ^∗∗∗∗^*p<*0.001 compared to NT-infected mice.

Our earlier findings that PIMT was readily recruited to the TRE-GRE region of *PCK1* promoter compared to the PPRE region in HepG2 cells^13^ prompted us to evaluate whether PIMT is recruited to cAMP-PKA signaling controlled CRE sites of *Pck1* promoter (Figure S4A). Using chromatin immunoprecipitation (ChIP) followed by PCR, we found that PIMT was indeed recruited to CRE-region of *Pck1* promoter in lean mice (Figure S4B). More importantly, ChIP-qPCR analysis showed significant enrichment of PIMT at the CRE region but not at GRE-TRE and PPRE sites in the *Pck1* promoter (Figure S4C). Having observed that fasting/PKA signaling enhances PIMT protein level and enhances recruitment to the CRE region of *Pck1* promoter, we next investigated the impact of PIMT on PKA-regulated hepatic gluconeogenesis. Freshly isolated mouse primary hepatocytes were infected with lentiviral particles expressing two independent short hairpin RNA (shRNA) against *Tgs1*. shSCR (nonspecific, NT) infected cells were used as the control. Post 48h of infections, cells were treated with either glucagon or Forskolin, and hepatic glucose output was evaluated. As shown in Figure 3A, depletion of PIMT significantly reduced both basal (DMSO treated samples) and glucagon/forskolin-induced hepatic glucose output. Similar experiments with insulin and metformin treatments (anti-hyperglycemic drugs) showed overall no additional significant effect on hepatic glucose output (Figure 3B).

Furthermore, to explore the potential role of PIMT in hepatic glucose metabolism, lentivirus preparations of *Tgs1* shRNA or scramble shRNA were administered into C57BL/6 mice through tail vein injection.^17^ Subsequently, mice were nutritionally deprived for 8h, and the expression of gluconeogenic genes was assessed by qPCR. As shown in Figure S4D, the expression of *Pck1* and *G6pc1* was depleted in both fed and fasting conditions in *Tgs1* shRNA-injected mice compared to shSCR-injected mice (NT). More importantly, fasting glucose levels were lower in PIMT shRNA-injected mice than in nonspecific control-injected mice (Figure 3C). After the oral glucose challenge (OGTT), systemic glucose disposal was significantly enhanced in mice injected with shRNA against *Tgs1* compared to mice receiving the shSCR injection (Figures 3D and 3E). To confirm the impact on insulin sensitivity upon PIMT depletion, we assessed the phosphorylation status of Akt and its upstream regulator p85 in the liver lysates of shPIMT-injected mice (Figures 3F and 3G). The expression level of p85 and the phosphorylation status of Akt were significantly enhanced upon acute knockdown of *Tgs1* in the liver. More importantly, the expression of PHLPP1, a well-documented phosphatase of Akt, was attenuated. Consistent with our previous observations,^12^ the expression of GLUT4 was also upregulated in PIMT-depleted hepatocytes. Expression of G6Pase served as an internal control for the fasting condition (Figures 3F and 3G). Acute depletion of PIMT also hampered hepatic gluconeogenic activity with decreased fasting glucose levels in the pyruvate tolerance test (PTT) Figures 3H and 3I. PIMT exerts this biological function by attenuating the expression of essential gluconeogenic genes, namely PEPCK, G6Pase, and PGC1α (Figures 3J and 3K). Collectively, these findings suggest that PIMT regulates systemic glucose tolerance mainly by governing hepatic glucose production in coordination with hepatic insulin sensitivity.

The observation that transient knockdown of PIMT suppresses hepatic gluconeogenic activity led us to wonder whether PIMT overexpression impacts systematic glucose clearance. To address this question, ectopic expression of PIMT was achieved by tail vein injection of lentiviral particles expressing PIMT. Reciprocal to our previous observations, systematic glucose disposal after OGTT was significantly enhanced in PIMT overexpressing mice (Figures S5A and S5B). Consistently, the protein level of p85 and the phosphorylation status of Akt were reduced considerably (Figures S5C and S5D). Expression of PHLPP1 was noted to be enhanced while the protein levels of glucose transporter GLUT4 were reduced. Expression levels of G6Pase were also noted to be robustly enhanced upon PIMT overexpression (Figures S5C and S5D). Similarly, upon the challenge of mice with PTT, the glucose production was significantly enhanced (Figures S5E and S5F) with increased expression of key gluconeogenic markers; PCK1, G6Pase, and PGC1α (Figures S5G and S5H). The above observations unambiguously establish PIMT as a potent regulator of hepatic glucose metabolism.

### PKA phosphorylates PIMT

Given the strong impact of PIMT expression on liver glucose metabolism, we reasoned that PIMT activity might also be directly regulated by PKA-mediated phosphorylation. We performed computational analysis to identify the potential phospho-acceptor sites of human PIMT. Such analysis revealed two PKA consensus phosphorylation sites (Ser^656^, Ser^851^) (Figures S6A and S6B). Besides, the Ser^656^ phospho-acceptor site of PIMT was evolutionarily conserved from yeast (*S*. *cerevisiae*), while Ser^851^ was reported to be present only in higher vertebrates (Figures S6C and S6D). More importantly, both the sites showed high score prediction using multiple phosphosite evaluators (Figure S6E, Motifscan, DISPHOS, Netphos, and Netphos kinase).

**Figure 4:**
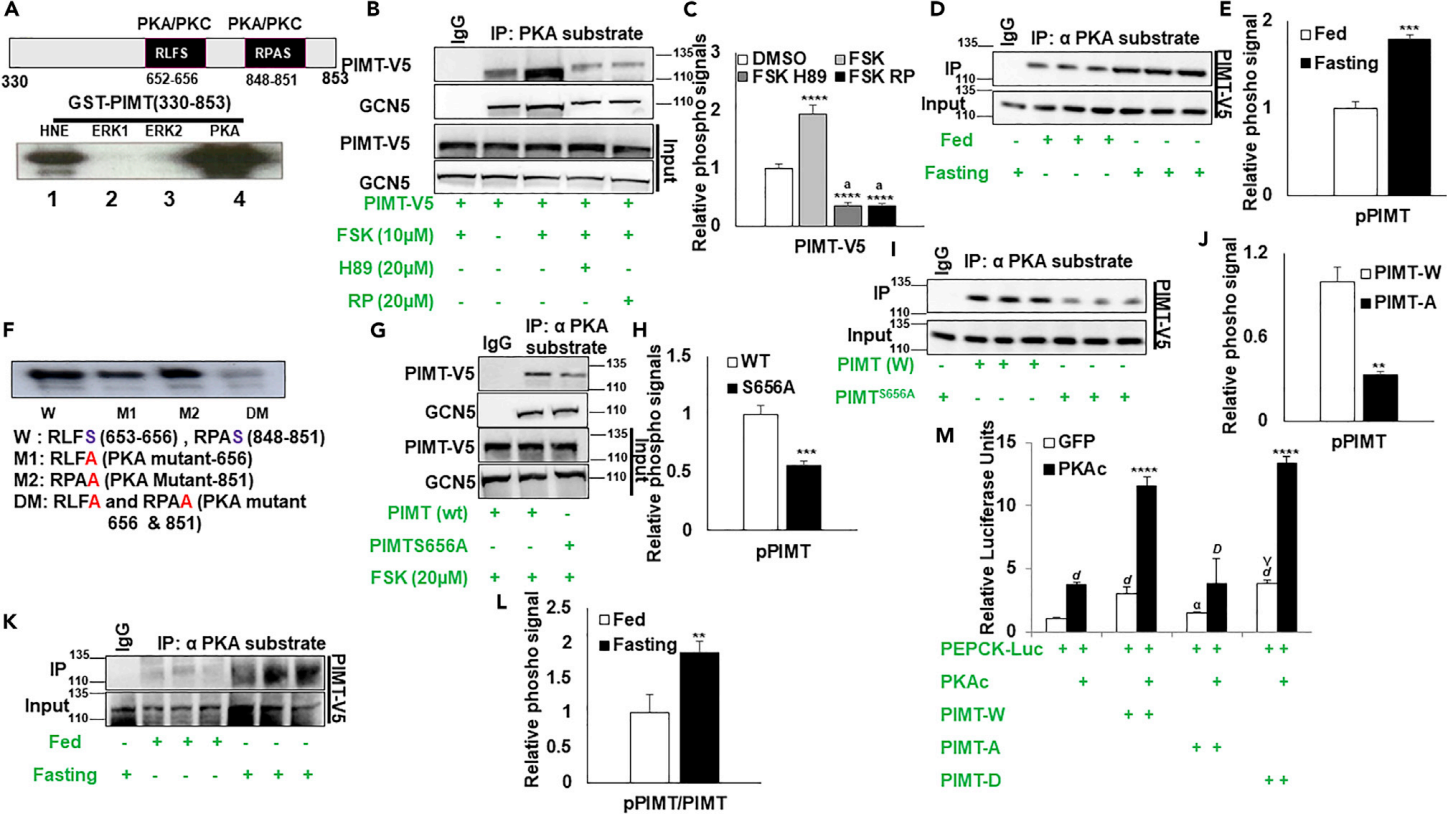
PIMT is phosphorylated by PKA at Ser^656^ (A) Sepharose bound GST fused PIMT-C was subjected to kinase reaction in the presence of HeLa nuclear lysate, constitutively active purified MAPKS (ERK1 and ERK2), or PKA. (B) Primary Hepatocytes infected with lentivirus expressing PIMT-V5 were exposed to Forskolin (10 μM) with or without H89 (20 μM) or RP (20 μM) for 1h and then subjected to IP with PKA substrate antibody followed by immunoblot with indicated antibodies. (C) Densitometric quantification of Figure 4B. The phosphorylation signals were normalized with their corresponding input. Numerical data are expressed as mean ± SD. Statistical analysis was performed using one-way ANOVA followed by Bonferroni’s post hoc test. ∗∗∗∗p *<* 0.001 compared to DMSO treated cells, ^a^*p<*0.001 compared to Forskolin treated cells. (D) Mice were infected with lentivirus expressing PIMT-V5 (n = 3). Post 7 days of infection, mice were fasted for 8h and liver lysates were subjected to IP with PKA substrate antibody followed by immunoblots with anti-V5. (E) Densitometric quantification of Figure 4D. The phosphorylation signals were normalized with the corresponding input signals. Numerical data are expressed as mean ± SD. Statistical analysis was performed using unpaired Student’s *t*-test (two-tailed) ^∗∗∗^*p<*0.005, versus the corresponding input. (F) Glutathione Sepharose beads bound GST-PIMT-C (W and mutants) were subjected to kinase assay with active and purified PKA. Double mutations at the PKA recognition site (RxxS, Ser^656^, and Ser^851^) abolished the phosphorylation of PIMT. (G) Primary hepatocytes isolated from female mice infected with either PIMT (W) or PIMT^S656A^ were treated with Forskolin for 4h and then subjected to IP with PKA substrate antibody followed by immunoblots with the defined antibodies (n = 4). (H) Densitometric quantification of Figure 4G. The phosphorylation signals were normalized with the corresponding input signals. Numerical data are expressed as mean ± SD. Statistical analysis was performed using unpaired Student’s *t*-test (two-tailed) ^∗∗∗^*p<*0.005, versus the corresponding input. (I) C57BL/6 male mice were tail-vein injected with lentivirus expressing PIMT (wt or S656A) (n = 3). Post 7 days of injection, mice fasted for 8h. Liver lysates were subjected to IP with PKA substrate antibody followed by immunoblots with the defined antibodies. (J) Densitometric quantification of Figure 4G. The phosphorylation signals were normalized with the corresponding input signals. Numerical data are expressed as mean ± SD. Statistical analysis was performed using unpaired Student’s t-test (two-tailed) ^∗∗∗^*p<*0.005, versus the corresponding input. (K) 8h fasted liver lysates subjected to IP with PKA substrate antibody followed by immunoblots with the defined antibodies (n = 3). (L) Densitometric quantification of Figure 4I. The phosphorylation signals were normalized with the corresponding input signals. Statistical analysis was performed using unpaired Student’s t-test (two-tailed) ^∗∗^*p<*0.01, versus the corresponding input. (M) HepG2 cells were transfected with the pGL3-PEPCK promoter and PIMT (W or mutants) encoding constructs with or without PKAc. Post-transfection cells were lysed, and luciferase readout was measured. The values were normalized with corresponding Renilla luciferase activity and expressed relative to PEPCK-Luc (unphosphorylated) (column 1), set to 1. Data are representative of 5 independent experiments and expressed as mean ± SD. Statistical analysis was performed using one-way ANOVA followed by Bonferroni’s post hoc test. ^*d*^*p<*0.001 vs PECK-Luc (unphosphorylated) ^∗∗∗∗^*p<*0.001 compared to PEPCK-Luc with PKAc transfected cells. ^γ^*p<*0.05, ^α^ p *<* 0.005 compared to PEPCK-Luc + PIMT (W) without PKAc, ^D^*p<*0.001 compared to PEPCK-Luc + PIMT (W) + PKAc, PIMT-W: PIMT wild type, PIMT-A: PIMT S656A mutant, PIMT-D: PIMT S656D mutant.

We performed *in vitro* kinase assay using purified GST fused fragment PIMT-C (330–853).^13^ *In vitro* kinase with purified PKA revealed that PIMT-C was robustly phosphorylated. Consistent with our previous report,^13^ both ERK1 and ERK2 failed to phosphorylate PIMT-C (Figure 4A). Phosphorylation of GST-PIMT-C by HeLa nuclear extract (HNE) served as the positive control. To evaluate whether PKA phosphorylates PIMT under cellular conditions, we overexpressed human PIMT in primary hepatocytes followed by treatment with PKA activator forskolin alone or in combination with PKA inhibitors H89 or RP. Post-treatment cells were lysed, and PKA substrates were enriched using the PKA-substrate antibody. The enriched samples were separated on SDS-PAGE and probed with indicated antibodies. As shown in Figure 4B, the treatment of cells with Forskolin enhanced the phosphorylation level of PIMT by ∼ 2-fold (Figure 4C). Co-treatment with either H89 or RP robustly reduced the phospho-signals of PIMT. GCN5 was used as an internal control for the assay.^18^ To evaluate whether PIMT is phosphorylated under *in vivo* conditions, transiently infected PIMT overexpressing mice were fasted for 8h and subjected to immunoprecipitation with PKA substrate antibodies. As shown in Figure 4D, the enrichment of PIMT-V5 was enhanced upon fasting (Figure 4E). Furthermore, recombinant PKA phosphorylated WT, mutant M1 (S656A), and M2 (S851A) but failed to phosphorylate double mutant confirming the authenticity of PKA phosphorylation sites (Figure 4F). As Ser^851^ residue is not conserved between humans and mice, the site was not further studied. Furthermore, mutation of evolutionary conserved Ser^656^ to alanine of human PIMT reduced PKA-dependent phospho-signals in forskolin-induced primary hepatocytes by ∼ 2-fold (Figures 4G and 4H). Likewise, phosphoSer^656^ PIMT signals were significantly reduced in Ser656Ala PIMT overexpressing mice upon fasting (Figures 4I and 4J). Importantly, we also observed that the phosphorylation of endogenous mouse PIMT was enhanced ∼2-fold in the liver lysates of fasting mice (Figures 4K and 4L). The phosphorylation status of GCN5 was minimally modified and served as the internal control (Figure 4G). Thus, we concluded that fasting-induced PKA activity enhanced the phosphorylation of PIMT at Ser^656^.

Having established that PIMT is a *bonafide* substrate of PKA, we next evaluated the functional relevance of the phosphorylation event. We transiently transfected cells with PEPCK-Luc and PIMT (wild-type and mutants) in the presence or absence of the catalytic subunit of the PKA complex (Figure 4M). The PEPCK-Luc activity was significantly stimulated by overexpression of either PKAc or PIMT (wt). Combined overexpression of PIMT and PKA displayed an inductive effect on luciferase activity. However, mutation of Ser^656^ to alanine significantly reduced both basal and PKAc-induced PEPCK-luciferase readout in HepG2 cells (Figure 4M). Supporting our previous findings,^13^ mutation of Ser^656^ to aspartate (which mimics phosphorylated PIMT Ser^656^ residue) induced PEPCK-Luc reporter activity comparable to wild-type PIMT, both under basal and PKAc-stimulated conditions (Figure 4M). Based on the above observations, it is likely that PKA-mediated PIMT phosphorylation may enhance its ability to promote PCK1 transcription and thus may play a vital role in controlling the gluconeogenic profile.

### PKA-mediated PIMT phosphorylation is a hepatic hyperglycemic driver *in vivo*

We previously reported that ectopic expression of PIMT augments hepatic glucose output in primary rat hepatocytes in MAPK/ERK-dependent manner.^13^ To evaluate the impact of PKA-mediated phospho-modification of PIMT in regulating hepatic gluconeogenesis, we infected primary mouse hepatocytes with lentiviral particles expressing either wild-type PIMT or its mutants. GFP-infected cells were used as the internal control. After 48h of infection, cells were treated with either glucagon, Forskolin, insulin, or metformin and were subjected to glucose output assay. Consistent with our previous report,^13^ PIMT wild-type (PIMT-W) overexpression significantly enhanced hepatic glucose output (Figure S7A). However, PIMT^S656A^ (PIMT-A) failed to augment basal hepatic glucose production and robustly suppressed glucagon or forskolin-induced hepatic glucose output. Interestingly, ectopic expression of PIMT^S656D^ (PIMT-D) robustly increased the glucose output compared to both GFP-infected cells and PIMT wild-type infected cells. More importantly, treatment with either glucagon or Forskolin further augmented hepatic glucose production upon PIMT wild-type infection. Interestingly, the overexpression of phospho-mimetic PIMT robustly enhanced glucagon and forskolin-mediated glucose production. Remarkably, the phospho-mimetic mutant displayed significantly enhanced glucose production compared to wild-type PIMT upon stimulation with glucagon and Forskolin (Figure S7A). Similarly, cells treated with insulin showed a significant reduction in hepatic glucose output upon PIMT overexpression, which was further reduced upon PIMT^S656A^ overexpression. Intriguingly, treatment with metformin abolished the effect of PIMT (wild-type) overexpression on hepatic glucose production. Curiously, the phospho-mimetic mutant overexpressing cells displayed robust glucose output even under the influence of metformin and insulin (Figure S7B). Taken together, the above findings indicate that PIMT phosphorylation at Ser^656^ represents a major crosstalk event between glucagon, insulin, and metformin-controlled regulatory signaling cascades.

Next, we verified the importance of Ser^656^ phosphorylation in the PIMT-mediated regulation of gluconeogenic genes by qPCR. Ectopic expression of PIMT (PIMT-W) significantly enhanced *Pck1*, and *G6pc1* expression along with *Phlpp1*, while the expression of *Slc2a4* was significantly blunted. Ser656Ala mutation of PIMT (PIMT-A) completely abolished the ability of PIMT to modulate the expression of the aforementioned genes. In contrast, overexpression of the phospho-mimetic mutant of PIMT (PIMT-D) retained the impact of PIMT on gluconeogenic gene expression (Figure S7C). We also tested fasting glucose levels in PIMT overexpressed mice. As anticipated, mere overexpression of PIMT significantly enhanced the circulatory basal glucose level compared to GFP-infected mice upon short-term fasting. Additionally, mutation of Ser^656^ to alanine impaired the ability of PIMT to increase the fasting glucose, while the phospho-mimetic mutant of PIMT retained its competence to augment basal glucose level in mice (Figure S7D). Collectively the presence of a negative charge on Ser^656^ residue is imperative in regulating PIMT-driven liver glucose homeostasis.

**Figure 5:**
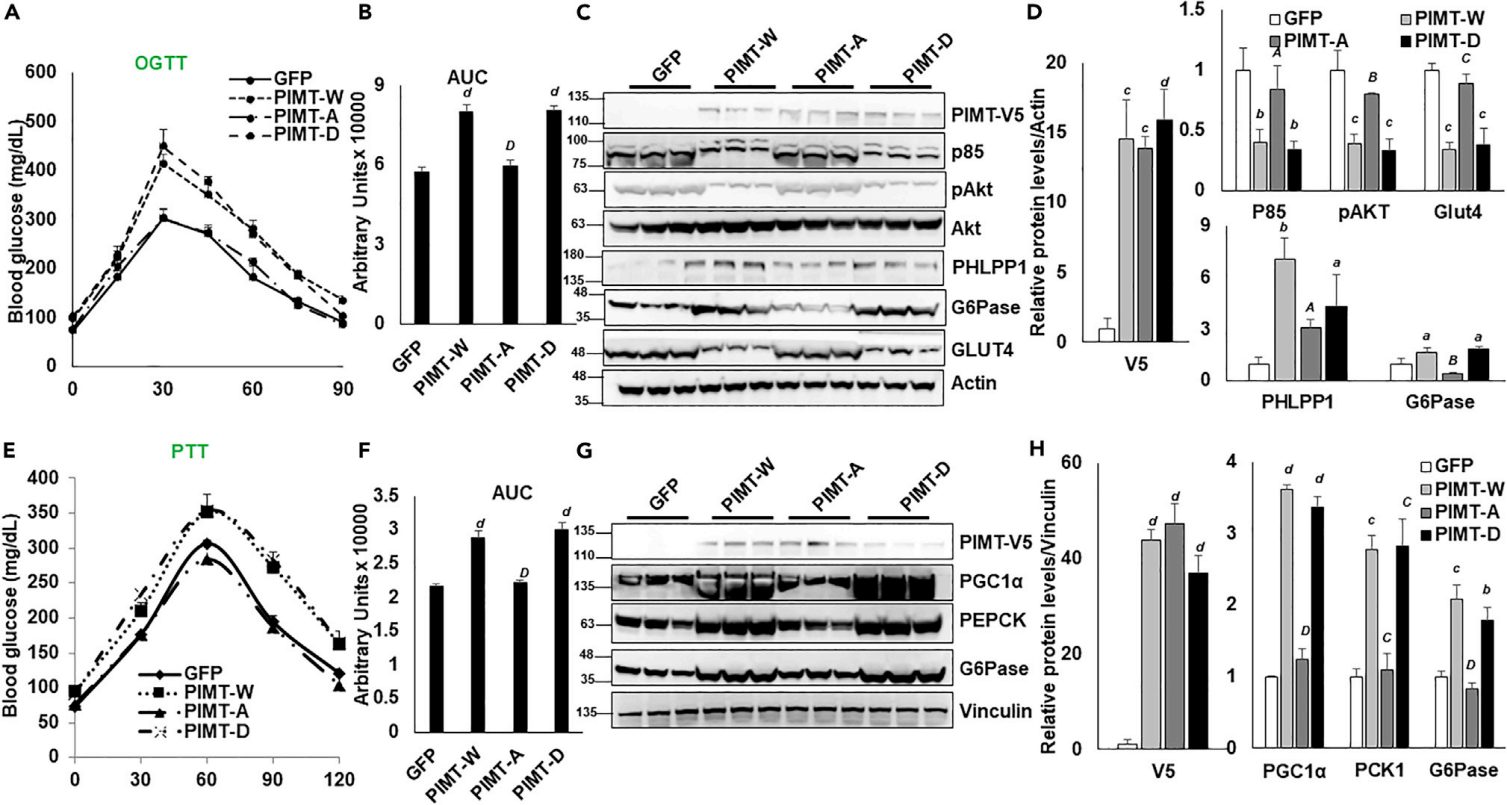
Phosphorylation of PIMT hampers liver glucose homeostasis (A) Oral Glucose tolerance test (OGTT) in C57BL/6J mice expressing GFP or PIMT (W or mutants) in the liver (n = 5). (B) The area under the curve for Figure 5A. Numerical data are expressed as mean ± SD. Statistical analysis was performed using one-way ANOVA followed by Bonferroni’s post hoc test. ^*d*^*p<*0.001 compared to GFP-infected mice, ^*D*^*p<*0.001 compared to PIMT-W-infected mice. (C) Immunoblot analysis of the liver lysates using the indicated antibodies. (D) Densitometric quantification of Figure 5C. Values were normalized with the corresponding loading control, actin. Numerical data are expressed as mean ± SD. Statistical analysis was performed using one-way ANOVA followed by Bonferroni’s post hoc test. ^*a*^*p<*0.05, ^*b*^*p<*0.01, ^*c*^*p<*0.005, ^*d*^*p<*0.001 compared to GFP-infected mice, ^*A*^*p<*0.05, ^*B*^*p<*0.01, ^*C*^*p<*0.005 compared to PIMT-W-infected mice. (E) Pyruvate tolerance test (PTT) in C57BL/6J mice expressing GFP or PIMT (W or mutants) in the liver. (F) The area under the curve for Figure 5E. Numerical data are expressed as mean ± SD. Statistical analysis was performed using one-way ANOVA followed by Bonferroni’s post hoc test. ^*d*^*p<*0.001 compared to GFP-infected mice, ^*D*^*p<*0.001 compared to PIMT-W-infected mice. (G) Immunoblot analysis of the liver lysates using the indicated antibodies. (H) Densitometric quantification of Figure 5G. Values were normalized with the corresponding loading control, Vinculin. Numerical data are expressed as mean ± SD. Statistical analysis was performed using one-way ANOVA followed by Bonferroni’s post hoc test. ^*b*^*p<*0.01, ^*c*^*p<*0.005, ^*d*^*p<*0.001 compared to GFP infected mice, ^*C*^*p<*0.005, ^*D*^*p<*0.001 compared to PIMT-W-infected mice. PIMT-W: PIMT wild type, PIMT-A: PIMT S656A mutant, PIMT-D: PIMT S656D mutant.

To gain insights into the physiological significance of PKA-mediated PIMT phosphorylation on systematic glucose control, we subjected PIMT (wild-type and mutants) overexpressing mice to OGTT. Surprisingly, the hypoactivation state of PIMT (Ser656Ala) in the liver of lean mice abolished the PIMT-mediated repressive impact on systematic glucose clearance and improved insulin sensitivity (Figures 5A and 5B). In contrast, the hyperactivation state of PIMT (Ser656Asp) retained the hyperglycemic properties of PIMT-driven gluconeogenesis (Figures 5A and 5B). Characterizing the effect on insulin signaling cascade, S656A mutation blunted PIMT-dependent suppression of the glucose transporter GLUT4 and p85 expression and its downstream phosphorylation of Akt (Figures 5C and 5D). On the other hand, expression of PHLPP1 and G6pase dwindled compared to PIMT (w) overexpressing mice (Figures 5C and 5D). In contrast to the S656A mutation, S656D decreased GLUT4 and p85 expression and phosphorylated Akt levels, almost comparable to PIMT (wt). Protein levels of PHLPP1 and G6Pase were similar to PIMT (wt) overexpression (Figures 5C and 5D). Additionally, animals with hepatic PIMT^S656A^ expression exhibited significantly reduced hyperglycemic response to pyruvate, while expression of aspartate mutant promoted glucose *de novo* biosynthesis from pyruvate (Figure 5E). Mechanistically, the hypophosphorylation status of PIMT failed to induce the gluconeogenic drivers of *de novo* synthesis of glucose (Figures 5E and 5F). In contrast, the hyperphosphorylation status of PIMT resulted in impaired glucose tolerance (Figures 5F and 5G). These results further demonstrate that PKA-dependent hyperactivation of PIMT acts as a critical driver in the dysregulation of mice’s glucose homeostasis.

### PKA regulates PIMT-Ep300 transactivation activity through phosphorylation

**Figure 6:**
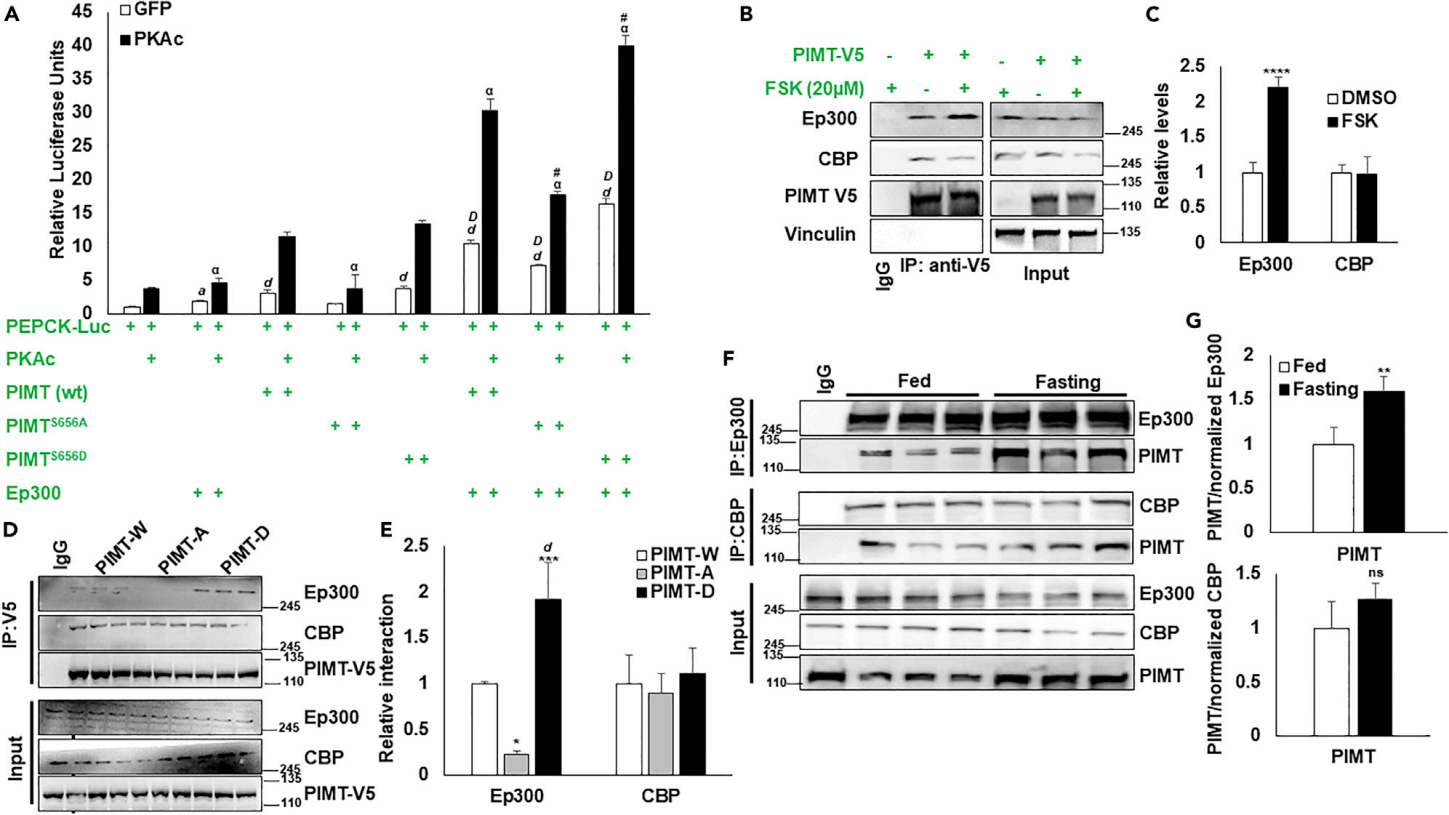
PIMT enhanced Ep300 coactivation activity (A) HepG2 cells were transfected with pGL3-PEPCK-Luc promoter along with Ep300, PIMT (W and mutants) in the presence or absence of PKAc. Post 36h of transfection, cells were lysed, and luciferase signals were quantified. Renilla luciferase signals were used as an internal control. The values were normalized with corresponding Renilla luciferase activity and expressed relative to PEPCK-Luc (unphosphorylated) (column 1), set to 1. Data are representative of 5 independent experiments and expressed as mean ± SD. Statistical analysis was performed using one-way ANOVA followed by Bonferroni’s post hoc test. ^a^*p<*0.05, ^d^*p<*0.001 vs PECK-Luc (unphosphorylated) ^α^*p<*0.001 compared to PEPCK-Luc + PIMT + PKAc transfected cells, ^D^*p*<0.001 compared to PEPCK-Luc + PIMT (W) without PKAc, ^#^*p<*0.001 compared to PEPCK-Luc + PIMT (W)+ Ep300 + PKAC. (B) Primary hepatocytes isolated from female mice infected with either PIMT (w) or GFP were treated with Forskolin for 4h and then subjected to IP with anti-V5 antibody followed by immunoblots with the defined antibodies (n = 4). (C) Densitometric quantification of Figure 6B. The signals were normalized with the corresponding input signals and neutralized with the PIMT-V5. DMSO-treated samples were set to 1. Data are representative of 3 independent experiments and expressed as mean ± SD. Statistical analysis was performed using unpaired Student’s t-test (two-tailed) ^∗∗∗∗^*p<*0.001, versus the DMSO, treated cells. (D) Primary hepatocytes isolated from female mice infected with either PIMT (W or mutants) (n = 3). Post-infection (48h), cells were lysed and subjected to IP with anti-V5 antibody followed by immunoblots with the defined antibodies. (E) Densitometric quantification of Figure 6D. The signals were normalized with the corresponding input signals and neutralized with the PIMT-V5 (W) set to 1. Numerical data are expressed as mean ± SD. Statistical analysis was performed using one-way ANOVA followed by Bonferroni’s post hoc test. ^∗^*p<*0.05, ^∗∗∗∗^*p<*0.001 compared to PIMT(W) infected cells. (F) 8h fasted liver lysates were subjected to IP with anti-CBP or anti-Ep300 antibodies followed by immunoblots with the defined antibodies (n = 3). (G) Densitometric quantification of Figure 6F. The interaction signals were normalized with the corresponding enriched protein signals. Statistical analysis was performed using unpaired Student’s *t-*test (two-tailed) ^∗∗^*p<*0.01, versus the fed liver lysates.

To determine the molecular mechanisms underlying PKA-mediated and PIMT-dependent enhancement of *de novo* glucose synthesis at the chromatin level, we investigated the possibility of Ep300 and CBP in coordinating PIMT-orchestrated gene regulation. Ep300 and CBP are crucial regulators of hepatic homeostasis through their acetyltransferase and transcriptional co-activator activities. During fasting, cAMP-PKA mediated phosphorylation of CREB at Ser^133^ recruits Ep300/CBP to CRE-containing genes, including *PCK1* and *G6PC1*.^19^^,^^20^ We previously reported that PIMT interacts and colocalizes with CBP and Ep300 in the nucleus.^9^ To evaluate the association between PIMT, Ep300, and CBP in PKA-driven gluconeogenic program, we transfected HepG2 cells with a luciferase reporter driven by *PEPCK* promoter together with either Ep300 or CBP and PIMT (wild-type and mutants) in the presence or absence of PKAc. PEPCK Luciferase activity was observed to be significantly enhanced upon the transfection of either Ep300 or PIMT wild-type and PIMT^S656D^, but not with PIMT^S656A^ (Figure 6A). The luciferase activity was further intensified in the presence of PKAc. Interestingly, co-expression of Ep300 and PIMT further amplified (∼3-fold more than PIMT (wt) alone and ∼5-fold more than Ep300 alone) the luciferase readings; furthermore, the activity of PEPCK promoter was noted to be robustly enhanced in the presence of PKAc, suggesting that cAMP-PKA mediated cascade exerts a positive impact on Ep300-PIMT driven gene regulation. However, overexpression of the phospho-deficient mutant PIMT in conjunction with Ep300 displayed a marked reduction in the luciferase readout. More importantly, the phospho-mimetic mutant of PIMT induced a significant increase in promoter activity compared to Ep300-PIMT (wt) in the presence and the absence of PKAc (Figure 6A). However, to our surprise, when we performed similar experiments with CBP, there was no positive impact on PEPCK promoter activity upon co-expression of CBP or PIMT either in the presence or absence of PKAc (Figure S8), suggesting that PKA-mediated phosphorylation of PIMT may enhance Ep300, but not CBP, driven hepatic gluconeogenesis. In support of this hypothesis, we treated primary hepatocytes overexpressing PIMT-V5 and evaluated its complex formation with acetyltransferases. As shown in Figure 6B, exposure to Forskolin facilitated the complex formation between Ep300 and PIMT while interaction with CBP was minimal altered (Figures 6B and 6C). Furthermore, we found that the mutation of PIMT S656A markedly reduced the immunoprecipitation of Ep300. In contrast, phosphomimic mutation further enhanced the co-immunoprecipitation, confirming the effect of phosphorylation on PIMT-Ep300 interaction in lean mice. The interaction of CBP was minimally altered upon PIMT mutations (Figures 6D and 6E). To further ascertain our observations at endogenous protein levels, we performed co-immunoprecipitation with either Ep300 or CBP in the fasted mice liver lysates. As expected, there was a significant enrichment of PIMT upon Ep300 pull down in lysates from the liver of fasted mice. However, the endogenous interaction of PIMT and CBP was least affected by the nutritional challenge (Figures 6F and 6G). These results demonstrate that phosphorylation at Ser^656^ of PIMT is an important event leading to enhanced tethering with Ep300 in regulating hepatic gluconeogenesis.

### Suppression of PIMT ameliorates diabetes

We also examined the phosphorylation status of PIMT at Ser^656^ in the liver of diabetic mice. Phosphorylation of PIMT (Figures S9A–S9D) and the expression of PIMT (Figures 1A–1F) were enhanced in the liver of *ob/ob* mice and wild-type fed on HFD. The interaction of PIMT with Ep300, but not CBP, was also increased in the liver of diabetic mice (Figures S9E–S9H). Such changes (PIMT phosphorylation and its interaction with Ep300) are consistent with the enhancement of gluconeogenesis which prompted us to evaluate whether PIMT contributes to promoting hepatic gluconeogenesis in these animals. To test this hypothesis, we injected lentiviral particles expressing two independent shRNA against *Tgs1* in diabetic mice. shSCR (NT) served as an internal control. Post-injection, mice were sacrificed, and the expression of gluconeogenic genes was quantified by qPCR. Consistent with our previous observation, depletion of PIMT significantly reduced *Pck1*, *G6pc1*, and *Phlpp1* transcript levels, while the expression of *Slc2a4* was enhanced (Figures S10A and S10B). Next, we performed ChIP assays to study whether PIMT was directly recruited to the *Pck1* promoter. Consistent with our earlier publication and above mention data, PIMT abundantly accumulated on the *Pck1* promoter. Strengthening our observations, accumulation of PIMT was also observed on *G6pc*, *Phlpp1*, and *Slc2a4* in the liver of insulin-resistant mice models (Figures S10C and S10D), compared to their corresponding control mice. More importantly, suppression of PIMT activity significantly lowered the fasting blood glucose in the diabetic mice models (Figures S10E and S10F), demonstrating that the regulatory function of PIMT in the expression of a specific subset of genes, especially encoding the gluconeogenic pathway, is vital in driving hyperglycemic conditions in insulin resistance.

**Figure 7:**
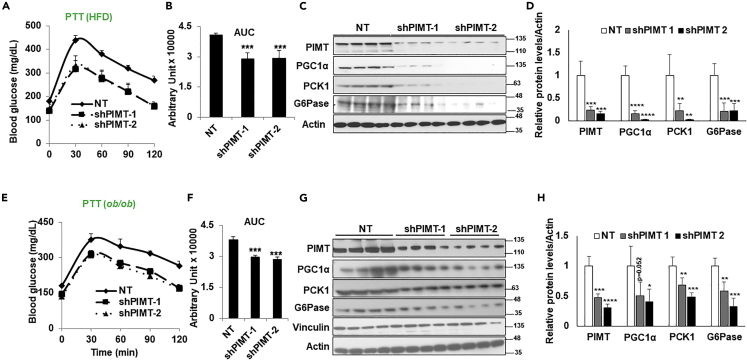
Hepatic PIMT depletion improves blood glucose levels in diabetic mice (A and E) Pyruvate tolerance test in HFD (A) or OB (E) mice expressing shSCR (NT) or two independent shRNA against *Tgs1* in the liver (n = 4). (B and F) The area under the curve for Figures 7A and B or Figures 7E and F. Numerical data are expressed as mean ± SD. Statistical analysis was performed using one-way ANOVA followed by Dunnett’s post hoc test. ^∗∗∗^*p<*0.005 compared to NT-infected mice. (C and G) Immunoblot analysis of the liver lysates using the indicated antibodies. (D and H) Densitometric quantification of Figures 7C and 7G. Values were normalized with the corresponding loading control, actin. Numerical data are expressed as mean ± SD. Statistical analysis was performed using one-way ANOVA followed by Dunnett’s post hoc test ^∗^*p<*0.05, ^∗∗^*p<*0.01, ^∗∗∗^*p<*0.005, ^∗∗∗∗^*p<*0.001 compared to NT-infected mice.

**Figure 8:**
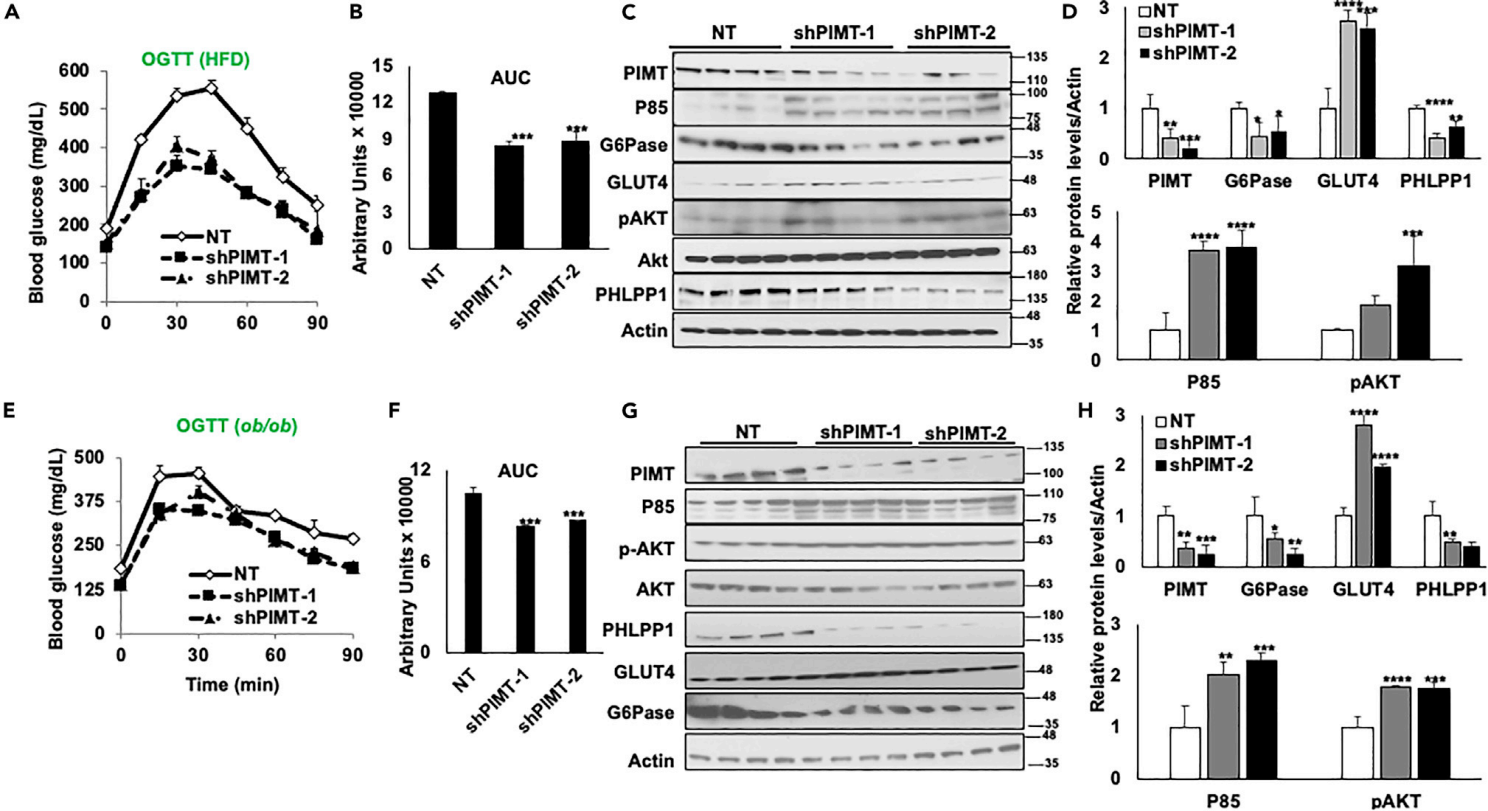
Hepatic PIMT depletion hampers *de novo* glucose synthesis in diabetic mice (A and E) Oral glucose tolerance test in HFD (A) or OB (E) mice expressing shSCR (NT) or two independent shRNA against *Tgs1* in the liver. (B and F) The area under the curve for Figures 7A and B or Figures 7E and F. Numerical data are expressed as mean ± SD. Statistical analysis was performed using one-way ANOVA followed by Dunnett’s post hoc test. ∗∗∗p *<* 0.005 compared to NT-infected mice. (C and G) Immunoblot analysis of the liver lysates using the indicated antibodies. (D and H) Densitometric quantification of Figures 7C and 7G. Values were normalized with the corresponding loading control, actin. Numerical data are expressed as mean ± SD. Statistical analysis was performed using one-way ANOVA followed by Dunnett’s post hoc test. ^∗^*p<*0.05, ^∗∗^*p<*0.01, ^∗∗∗^p*<*0.005, ^∗∗∗∗^*p<*0.001 compared to NT-infected mice.

Having observed a robust decrease in gluconeogenic genes followed by a significant decrease in blood glucose level, we next challenged the mice with pyruvate with a presumption that PIMT depletion will remarkably reduce the *de novo* glucose synthesis. As anticipated, the acute suppression of PIMT significantly hampered the non-carbohydrate conversion to glucose in the liver of diabetic mice models (Figures 7A, 7B, 7E, and 7F). In support of this, levels of gluconeogenic proteins PEPCK, G6Pase, and PGC1α were diminished in PIMT depleted liver lysates (Figures 7C, 7D, 7H, and 7H). To further ascertain the pathological impact of hepatic PIMT on glucose homeostasis in insulin-resistant mice, we challenged the mice with oral glucose (OGTT). OGTT represents one of the most widely accepted tests to determine the glucose-intolerant capacity and diabetes in genetically engineered or diet-induced mouse models. In harmony with the above-described observations, shRNA-mediated depletion of PIMT significantly improved the glucose clearance capacity from the peripheral circulation in diabetic mice models (Figures 8A, 8B, 8E, and 8F). Furthermore, the levels of the insulin signaling component, p85, were upregulated, followed by enhanced activity of Akt (pAkt) (Figures 8C, 8D, 8G, and 8H). In contrast, the expression of PHLPP1, which hampers the insulin signaling cascade, was reduced in the obesity mice models compared to shSCR-infected mice. Additionally, the expression of glucose transporter was also found to be upregulated. In accordance with our observations, the expression of G6Pase was reduced upon PIMT depletion (Figures 8C, 8D, 8G, and 8H). Collectively, the data establish that the disruption in PIMT’s activity (either expression or phosphorylation) attenuates gluconeogenesis and ameliorates hyperglycemia by improving hepatic insulin sensitivity and suppressing glucose biosynthesis from non-carbohydrate sources.

## Discussion

Our work collectively establishes a unique governing network that delineates the complex obesogenic diet-induced insulin resistance, providing feasibility and mechanistic basis for therapeutic implications for reversing over-nutrition-associated metabolic syndrome. Our studies identify the PKA-PIMT phosphorylation switch in the hepatocytes as a regulatory mechanism to ensure robust glucose production. Dysregulation of the PKA-PIMT axis, low-grade chronic inflammation, and a high-fat/western diet perpetuates insulin resistance development. Interestingly, this signaling intermediate, PIMT, may be targeted to reverse or attenuate diet-associated insulin resistance.

Short-term fasting has a dominant role in sustaining serum glucose levels, an essential physiological process that is hijacked in metabolic diseases, including Type 2 Diabetes Mellitus. The importance of glucagon in the fasting-associated glucose secretion and pathogenesis of diabetes is well characterized. Increased glucagon signaling leads to dysregulated glucose homeostasis, whereas a decrease in glucagon action improves glycemic index in diabetes independent of insulin sensitivity.^21^^,^^22^^,^^23^^,^^24^^,^^25^^,^^26^ Transcriptional dysregulation of the hepatic gluconeogenic signature genes due to changes in the hormonal level in the portal vein is a hallmark of hepatic insulin resistance.^2^^,^^27^ PCK1 and G6Pase are the rate-limiting enzymes of *de novo* glucose synthesis and, therefore, robustly regulated at the transcript level by a plethora of transcription factors/nuclear receptors and co-regulators. Despite this deep understanding of the complex regulatory network, the limited application in clinics has motivated the research community to identify novel broad actionable targets. Even though several protein players were recently shown to play an essential role in fueling gluconeogenic transcription, we have focused on the third generation of transcriptional regulators, PIMT.

Our previous study showed that pPIMT (Ser^298^) is critical for enhancing Med1-mediated hepatic gluconeogenesis.^13^ Modulating PIMT expression (either upregulation or downregulation) in primary hepatocytes directly impacted the gluconeogenic gene expression and, thus, glucose production.^13^ Similarly, overexpression of PIMT also enhanced hepatic gluconeogenic gene expression in the fasted state and glycemia after fasting or pyruvate administration. Likewise, depletion of PIMT in the liver of mice revealed reduced glycemia or pyruvate clearance due to a significant reduction in hepatic gluconeogenic expression. In addition, assessing the impact of PIMT expression on the insulin signaling cascade, we noted that PIMT negatively regulates insulin sensitizers in part by promoting the expression of PHLPP1, an established intracellular Akt,^28^ and AMPK inhibitor.^16^ Thus, PIMT has been proposed to function as a negative regulator of insulin signaling, consequently promoting hepatic gluconeogenesis, prompting us to evaluate its expression in insulin resistance pathological models.

Consistently, PIMT expression (at protein and mRNA levels) was noted to be upregulated in nutritionally burdened and in *ob/ob* mice. Surprisingly, short-term fasting elevated endogenous hepatic PIMT protein levels with minimal impact on its transcript. Molecular dissection utilizing molecular, cellular, and *in vivo* approaches to such observations revealed that fasting-induced PKA signaling enhances the translational capacity of the *Tgs1*mRNA to enhance the protein content. Although the exact underlying mechanism is still unknown, it appears that the translation of *TGS1*mRNA was preferentially enhanced upon PKA activation. This is intriguing because PIMT regulates a panel of gluconeogenic genes at the transcript level; therefore, our results show that PIMT being regulated at the post-transcriptional level by fasting-induced signaling may provide a quick activation for the transcriptional machinery during the glucose shortage. However, further studies will be necessary to demonstrate the cellular partners involved in the selective up-regulation of PIMT mRNA for enhanced translation during short-term fasting in hepatocytes.

**Figure 9:**
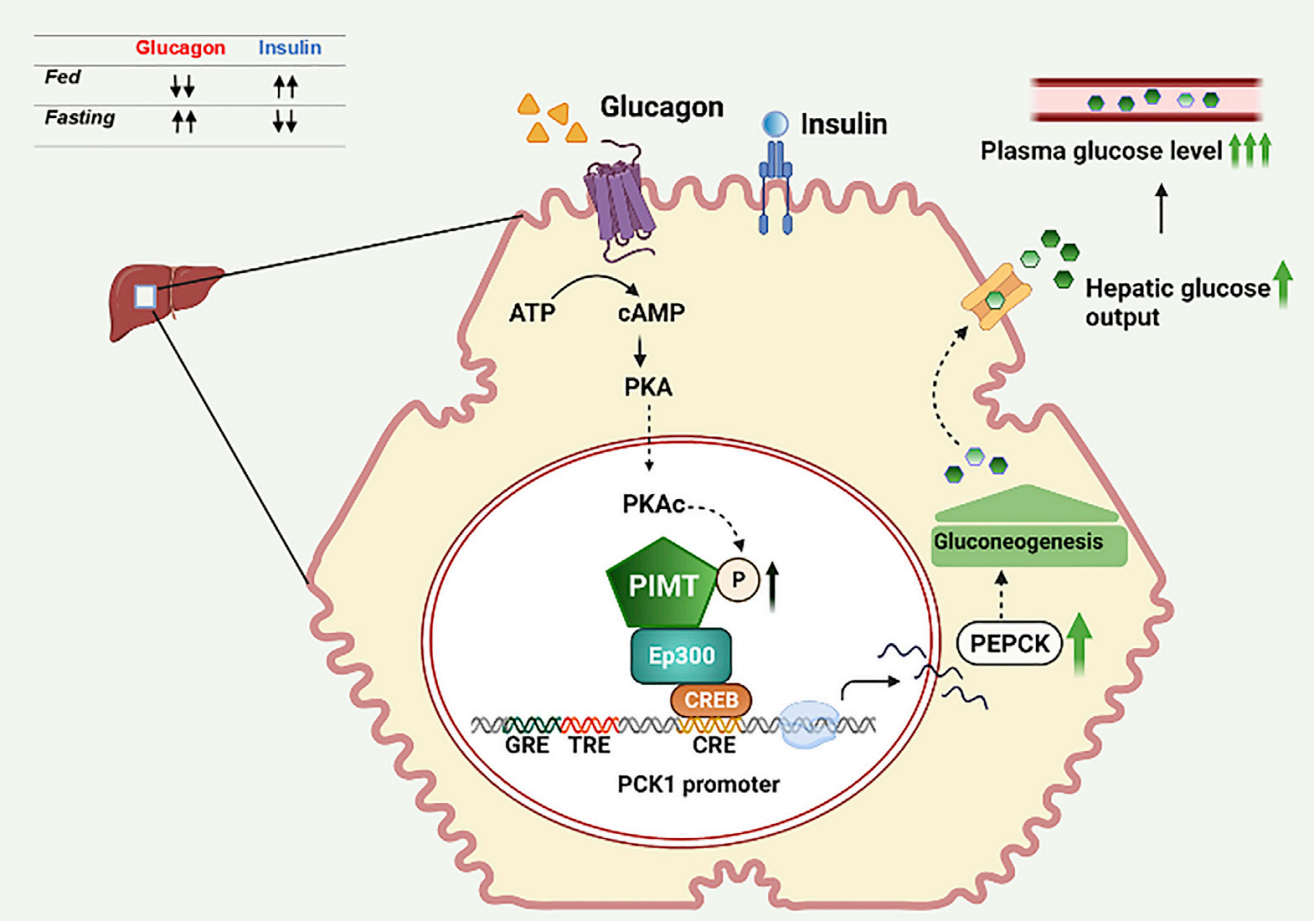
PKAc-driven PIMT activity regulates hepatic glucose metabolism Protein levels of PIMT are elevated in the liver upon short-term fasting and in HFD & *ob/ob* mice. The activity of PIMT is finely regulated in the liver in response to changes in nutritional availability. During fasting, glucagon-induced PKAc enhances the protein levels of PIMT in hepatocytes. Notably, PKAc also phosphorylates PIMT which augments the expression of gluconeogenic genes resulting in enhanced glucose production. Curtailing the expression of PIMT or hampering the PKAc-PIMT axis alleviates the glucose burden in obese and diabetic rodents.

The impact of the post-transcriptional/translation regulation of PKA signaling on PIMT expression was only evident in wild-type mice upon fasting but not in mice with insulin resistance. This prompted us to evaluate whether the PKA pathway regulates PIMT at the post-translational level. In this regard, using conventional tools, we established that PIMT is a PKA substrate and PKA phosphorylates PIMT at Ser^656^ residue. Ectopic expression of PIMT^S656D^ displayed an enhanced glycemic index under both fasting and pyruvate clearance. However, forced expression of PIMT^S656A^ in the liver failed to elicit any alterations in the glucose levels compared to PIMT (wt and asp mutant), revealing that PKA-mediated PIMT phosphorylation enhances the PIMT’s transcriptional functionality. Thus, the phosphorylation of PIMT at Ser^656^ by PKA stimulation may represent an additional mechanism whereby fasting/PKA signaling maintains basal gluconeogenic program in the liver and favor quick response during serum glucose depletion.^19^ The dual regulation of PIMT in hepatocytes by PKA signaling seems logical, given that protein is critical in pathophysiological conditions for glucose homeostasis^12^^,^^13^^,^^14^ (Figure 9).

The predominant regulation of gluconeogenesis by fasting/PKA signaling via the CREB-CBP-CRCT2 complex is a crucial signaling pathway.^15^^,^^29^ Wondisford and colleagues' recent work suggests that Ep300 is perhaps responsible for basal hepatic gluconeogenesis and can be active even in the postprandial state when insulin levels are high.^19^ Likewise, PIMT was noted to be recruited to the *Pck1*promoter under basal conditions in primary mouse hepatocytes and liver. This observation provoked to study the impact of CBP and/or Ep300 on PKA-PIMT-driven *PCK1* expression. Our experiments suggest that PIMT works cooperatively with Ep300, but not with CBP, to activate the gluconeogenic program in fasting. In generalizing the interactions between CBP, Ep300, and PIMT in the overall regulation of hepatic glucose output, we have observed that PIMT-Ep300 can promote PKA-driven hepatic glucose synthesis, whereas CBP-PIMT was minimally modified. However, it will be interesting to study CBP-CRTC2-PIMT crosstalk, if any, in liver glucose metabolism.^9^^,^^30^ Data obtained in this study are in striking contrast with our previous publication,^9^ further justifying the dynamic impact of PIMT in regulating co-activator-driven transcriptional regulation in a context-dependent manner.

Our findings add an additional level of complexity to the regulation of *de novo* glucose biosynthesis. This raises several questions. Does re-feeding/insulin signal impact PIMT expression and/or transcriptional activity? What is the impact of clinically relevant anti-hyperglycemic drugs such as metformin on PIMT’s activity? Moreover, can any generalization be made on the fluidity of PIMT’s covalent phospho-modification in the regulation of hepatic gluconeogenesis? Regarding the first question, our preliminary study does direct that insulin/feeding has a direct impact on PIMT protein levels, in part, by regulating its 3′UTR activity (Figure S12). However, a more detailed analysis is required for a holistic understanding of the PIMT expression and nutritional status. As for the second question, we are currently pursuing additional research to assess the molecular underpinnings of anti-hyperglycemic drugs such as insulin and metformin on PIMT’s activity. Initial observations from PIMT overexpressing primary hepatocytes revealed a significant decrease in hepatic glucose output upon insulin or metformin treatment. However, to our surprise, ectopic expression of PIMT^S656D^displayed a trivial decrease in glucose production compared to WT protein, revealing that PKA-mediated phosphorylation may hamper the anti-hyperglycemic effect on glucose production. Concerning PIMT post-translational phospho-modifications, we have observed that phosphorylation at Ser^298^ and Ser^656^ are critical for PIMT functionality under ERK^12^^,^^13^ and PKA signaling, respectively. Integrating these observations, the effect of each phosphorylation event on hepatic gluconeogenesis will depend upon the external clues and the dominant co-regulator contributing to the maintenance of gluconeogenesis.

Depleting PIMT in the liver of mice fed on an obesogenic diet and type 2 diabetes mellitus suppressed gluconeogenic gene expression and improved glycemic index. Furthermore, ectopic expression of PIMT^S656A^ failed to alter the glycemic index in wild-type mice, compared to PIMT-wt-expressing mice. This observation suggests that the inhibition of PIMT or culminating PKA-PIMT phosphorylation may ameliorate diabetes. Disruption of the PKA-PIMT-Ep300 axis (which is achieved by PIMT depletion) also suppressed gluconeogenesis, suggesting that this module is a promising pharmacological target for treating obesity and type 2 diabetes.

### Limitations of the study

We observed a significant reduction in the expression of the gluconeogenic genes in primary hepatocytes upon PIMT depletion. Consistently, circulatory glucose levels were noted to be reduced in diabetic mice models. Although the function of the PIMT in the regulation of gluconeogenic genes is clear, the intramolecular machinery regulating the PIMT expression is still not well established. Also, our observation that starvation-induced PKA signaling augments the PIMT protein synthesis requires deeper dissection of the molecules associated with increasing the *Tgs1* transcript load on the translational apparatus machinery. Furthermore, the current study falls short of evaluating the direct inputs of Insulin and Metformin-induced signaling in regulating PIMT-driven hepatic glucose regulation.

## STAR★Methods

### Key resources table

**Table:** 

REAGENT/RESOURCE	SOURCE	IDENTIFIER
**Antibodies**		

Anti-PCK1	SantaCruz Technologies	sc-271029; RRID: AB_106110383
Anti-G6Pase	SantaCruz Technologies	sc-25840; RRID: AB_2107514
Anti-PCG1α	SantaCruz Technologies	sc-518025; RRID: AB_2890187
Anti-PIMT	Abcam	ab70559; RRID: AB_1269767
Anti-PHLPP1a	Bethyl	A300-661A; RRID: AB_2299551
Anti-pCREB	Cell signaling technology	#9198; RRID: AB_2561044
Anti-PKAc	Cell signaling technology	#4782; RRID: AB_2170170
Anti-PKAc substrate	Cell signaling technology	#9624; RRID: AB_331817
Anti-Phospho Akt Sert473	Cell signaling technology	#4060; RRID: AB_2315049
Anti-Akt	Cell signaling technology	#4685; RRID: AB_2225340
Anti-P85	Cell signaling technology	#4257; RRID: AB_659889
Anti-GLUT4	Novus Biologicals	NBP1-49533
Anti-CBP	Cell signaling technology	#7425; RRID: AB_10949975
Anti-Ep300	SantaCruz Technologies	sc-48343; RRID: AB_628075
Anti-V5	ThermoFisher	R960-25; RRID: AB_2556564
Anti-Flag	Millipore Sigma	F1804-50UG; RRID: AB_262044
Anti-Actin	SantaCruz Technologies	sc-47778; RRID: AB_626632
Anti-Vinculin	Millipore Sigma	V9264-100UL; RRID: AB_10603627
Anti-Mouse HRP	SantaCruz Technologies	sc-516102; RRID: AB_2687626
Anti-Rabbit HRP	SantaCruz Technologies	sc-2357; RRID: AB-628497

**Chemicals, peptides, and recombinant proteins**		

SuperSignal™ West Pico PLUS Chemiluminescent Substrate	Thermo Fisher Scientific	34579
Rp-8-Br-cAMPS	Millipore Sigma	B2432
DMEM	Lonza	BE12-604F
Fetal bovine serum	Himedia	RM9955
Power Sybr green master mix	Thermo Fisher Scientific	4368577
PEI	Polyscience Inc	23966
H-89 dihydrochloride hydrate	Millipore sigma	B1427
MG-132, Ready Made Solution	Millipore sigma	M7449-1ML
60% high fat diet	Research Diets	D12492
RIPA buffer	Cell Signaling Technology	9806
TRIzol RNA isolation reagent	Thermo Fisher Scientific	15596026
MK2206	Selleck Chem	S1078
U0126	Selleck Chem	S1102
Care Touch Blood Glucose Meter	Amazon	CT210
Recombinant Human Active ERK1 Protein	RnD Systems	1879-KS-010
Recombinant Human Active ERK2 Protein	RnD Systems	1230-KS-010
Recombinant Human Active PKA C beta Protein	RnD Systems	268-KS-010

**Critical commercial assays**		

High-Capacity cDNA Reverse Transcription Kit	Thermo Fisher Scientific	4368813
Glucose colorimetric assay kit	Cayman	10009582
Site directed Mutagenesis kit QuikChange II	Agilient	200523

**Experimental models: Organisms/strains**		

Mouse: WT C57BL6/J	Indian Institute of Chemical Biology, Kolkata	In bred; RRID: MGI:2177676
Mouse: Lepob (*ob/ob*)	Jackson Laboratories	Purchased; RRID: MGI:5816460

**Experimental models: Cell lines**		

HepG2	ATCC	HB-8065; RRID: CVCL_0027
HEK293T	ATCC	CRL-3216; RRID: CVCL_0063
Primary Hepatocytes`	Freshly isolated	In house

**Oligonucleotides**		

PIMT_Fwd: GCCATCGACAGGTCAGGTAT	Integrated DNA Technologies IDT	N/A
PIMT_Rev; TGAACAGGATGTGCTTGCTC		N/A
PCK1_Fwd: AAGGAAAACGCCTTGAACCT		N/A
PCK1_Rev: GTAAGGGAGGTCGGTGTTGA		N/A
Mouse CRE-Fwd: ACCGTGCTGACCATGGCTAT		N/A
Mouse CRE-Rev: TGTGTTCCCAGAGGGAAGGC		N/A
Mouse GRE-Fwd: CCAGCTAACTCAGCAGGTACAGA		N/A
Mouse GRE-Rev: GGTGGCTGCTGGTTGTCAA		N/A
Mouse PPRE-Fwd: CTCTCTCCCATTGACTTCTCACTCAC		N/A
Mouse PPRE-Rev: GTGGCACTTGAGCAACAAGACC		N/A

**Recombinant DNA**		

pDonR201 (Wt and mutants)	PCR cloning, GW technologies	pcDNA-PIMT-Flag as template^13^
SFB-PIMT (Wt and mutants)	GW cloning	Behera et al. 2018^31^
pLenti-PIMT (wt and mutants)	GW cloning	Thermo Fisher (pLenti 6.3 kit) Catalog number: V53306
pLenti-PIMT-3'UTR	This study	Human 293T cDNA was used as the template
pGL3-PEPCK promoter	In lab cloning	Kapadia et al. 2013^13^
pLKO.1-shTsg1	Sigma Aldrich	TRCN0000090626, TRCN0000338134
psPAX2	Addgene	12260; RRID: Addgene_12260
pMD2.G	Addgene	12259; RRID: Addgene_12259
GST-PIMT-N (1-334)	In lab cloning	Kapadia et al. 2013^13^
GST-PIMT (330-853) (Wt and Mutants)	In lab cloning	Kapadia et al. 2013^13^
pCMVb-Ep300	Addgene	10717
pcDNA3β-FLAG-CBP-HA	Addgene	32908; RRID: Addgene_32908
pCalpha EV (PKA catalytic subunit Calpha)	Addgene	15310; RRID: Addgene_15310

**Softwares**		

GraphPad Prism 6	GraphPad Software	https://www.graphpad.com/scientific-software/prism/; RRID: SCR_002798
EndNote™ X8	Endnote	Product Details | EndNote; RRID: SCR_014001
LI-COR Image Studio Software	Licor Biosciences	Download Free Image Studio Lite for Western Blot Quantification (licor.com); RRID: SCR_015795

### Resource availability

#### Lead contact

Information and requests for resources and reagents should be directed to and fulfilled by the lead contact, Parimal Misra (parimalm@drils.org).

#### Materials availability

All materials used in this study are either commercially available or through collaboration, as indicated.

### Experimental model and subject details

#### Mice

C57BL/6 (B6) mice were fed a high-fat diet (60% fat calories, 20% protein calories, and 20% carbohydrate calories; Research Diets) or a normal chow diet *ad libitum*. In most assays, 8 weeks old mice (male and female) were fed with HFD for 12 weeks. All mice used in this study were maintained at 22°C in a 12/12 h light-dark cycle in a specific pathogen-free facility and given free access to food and water, and studies were conducted during the light cycle. Additionally, for lentivirus-mediated gain and loss of function, aged-matched wild-type and obese mice (loss of function study) were injected intravenously through tail vein with the indicated particles in figure legends. Post 4 days of injection, mice received booster injection. HFD-fed mice (12 weeks fed) were injected (intravenous) with lentivirus expressing shRNA against Tgs1 or scramble, followed by a booster injection on the 4th day. After 7 days of the injection, mice fasted for 8h (OGTT) or 16h (PTT) studies. For the HFD study, mice were on a high-fat diet during the complete study.

#### Study approval

All animal procedures were done according to the Animal Ethics Committee guidelines of the University of Hyderabad, Hyderabad (No: UH/IAEC/PB/2020–1/40) and Indian Institute of Chemical Biology, Jadavpur, Kolkata 700032(No: IICB/AEC/Meeting/July/2020/10). All animals were randomly assigned cohorts when used.

#### Glucose tolerance and pyruvate tolerance tests

For glucose tolerance tests, mice orally received one dose of glucose (2 g/kg body weight) after 8h of fasting. For pyruvate tolerance tests, mice were fasted for 16h and then i.p. injected with pyruvate (2g/kg body weight). Blood was drawn from the tail vein, and glucose levels were quantified with a hand-held glucometer (Care Touch Blood Glucose Meter).

#### Isolation of primary hepatocytes

Primary hepatocytes were isolated as described previously.^13^ Briefly, mice were infused with a calcium-free HEPES-phosphate buffer A (Calcium and magnesium-free PBS containing 0.2 μM EGTA pH 7.4) via the vena cava for ∼5 min. Observing the color of the liver changing to beige or light brown color, collagenase-containing buffer B (PBS with one mM magnesium and one mM calcium, 0.5mg/mL collagenase H) was perfused into the liver. For ∼5 min. Noticing the crack on the surface of the liver, perfusion was stopped immediately, and the liver was excised into ice-cold buffer A. Cells from digested livers were teased out, suspended in Buffer A, filtered through 100 μm cell strainer, and centrifuged at 100 × g for 5 min at 4°C. The pellets were washed with Buffer B (no collagenase) twice. The cells were cultured in complete DMEM with 20% FBS) on collagen-coated plates. The media was refreshed after overnight incubation.

#### Glucose output assay

After serum-starved for 6h, freshly isolated primary hepatocytes were washed twice and then exposed to glucose-free media (phenol-red and glucose-free DMEM containing 20mM sodium lactate, 2mM sodium pyruvate). Cell supernatants were collected 6h later and were subjected to glucose measurement using a Glucose (GO) assay kit (GAG0-20, Sigma, St. Louis, MO, USA). Cells were lysed, and protein concentration was determined using the Bradford reagent. The glucose output was normalized to cellular protein concentration and was expressed as arbitrary units.

### Method details

#### Cell culture, plasmids, transfection, and reagents

HEK-293T and HepG2 were procured from ATCC and maintained routinely in DMEM supplemented with fetal bovine serum (FBS) at 37^o^C with 5% CO2. Reagents were procured from Sigma unless otherwise mentioned. Cells were regularly passaged as per the manufacturer's guidelines. Exponentially growing HepG2 cells were treated with reagents at different concentrations and different time points, as mentioned in the corresponding figure legends. According to the manufacturer's instruction, the expression constructs SFB (S protein/FLAG/Streptavidin-binding peptide)-tagged PIMT and pLenti6.3-PIMT-V5 were cloned using Gateway recombination cloning technology (Thermo scientific). Expression construct of PKA catalytic subunit (Plasmid #15310), Ep300 (Plasmid #10717), CBP (Plasmid #10718) were procured from Addgene. pLKO.1- shPIMT and pLKO.1-SCR were procured from the Sigma RNAi consortium. PIMTS656Aand PIMTS656D mutants were generated using a Quick-change site- directed mutagenesis kit as per the manufacturer's instructions. Transient transfection with different expression constructs was performed using Polyethylenimine (Polysciences) following the manufacturer's instructions. Post 24 h of transfection, cells were harvested and processed for the downstream experiments.

HEK-293 T cells were seeded at ∼50% confluency for lentiviral production and transfected the following day with psPAX2 (Addgene #12260) and pMD2G (Addgene #12259) along with pLKO.1-SCR and pLKO.1-shPIMT (mouse) constructs. Virus containing medium was collected post 48 h and 72 h of transfection and was concentrated using Amicon Ultra−15,100 kDa centrifugal filters as per the manufacturer's instructions. Lentiviral particles expressing PIMT, and its mutant were generated using a Virapower lentiviral expression kit with transfection in 293FT cells. Viral particles were collected post 72h of injection and were concentrated using centricon as described above. The lentiviral particles used for the complete study expresses shRNA under the human U6 promoter.

For the promoter-luciferase assay, each transfection mix contained 700ng of reporter PEPCK promoter, 300ng of CBP/Ep300, 200ng of PKAc, and 100ng of renilla luciferase expression vectors along with 300ng of PIMT or PIMTS656A or PIMTS6565Dexpression constructs. Cells were lysed 30h after transfection with passive lysis buffer (Promega, Madison, WI, USA) and equal amounts of lysate and luciferase assay buffer (20mM tricine, 1.07mM magnesium carbonate, decahydrate, 0.1mM EDTA, 2.67mM MgSO4, 33.3mM DTT, 270μM co-enzyme A, 470μM d-Luciferin, 530μM ATP) were mixed. Luminescence values were recorded in Sirius Luminometer (Berthold Detection Systems GmbH, Pforzheim, Baden-Württemberg, Germany). The values were normalized to the co-transfected renilla luciferase activity (renilla luciferase assay buffer: 4μM coelenterazine, 50μM phenyl benzothiazole, 25mM sodium pyrophosphate, 15mM EDTA, 10mM sodium acetate, 500mM sodium sulfate, and 500mM sodium chloride). For 3'UTR Luciferase assay, 700 ng pLenti empty luciferase construct or pLenti3/UTR PIMT luciferase construct and 100ng of renilla luciferase expression vector were transfected in HepG2 cells. Where indicated, cells were co-transfected with the PKAc construct. Post transfections cells were treated with indicated reagents at the specified concentration (see figure legends), and luciferase was estimated using Sirius Luminometer (Berthold Detection Systems GmbH, Pforzheim, Baden-Württemberg, Germany).

#### Cell lysis, western blotting, and immunoprecipitation

Post-treatment cells were lysed with ice-cold 1X RIPA lysis buffer (50 mM TRIS pH 7.5, 150 mM NaCl, 0.1% SDS, 0.5% sodium deoxycholate, 1% Triton X-100, 1 mM EDTA, and 1 mM EGTA, 1 mM sodium orthovanadate, 1 mM sodium fluoride, 1× protease inhibitor (Sigma-Aldrich), phosphatase inhibitor cocktails #2 and #3 (Sigma-Aldrich), and 1 mM PMSF). Cells lysate was quantified using Bradford reagents, and an equal amount of protein was separated on an SDS-PAGE and probed with the antibodies described in key resources table. Densitometry analyses were performed using Image Studio (Licor Biosciences) and presented as target band signal intensity ratio to GAPDH/Actin/Vinculin band signal intensity. For immunoprecipitation, precleared 300 μg of cell lysates were incubated with either PKA substrate antibody (2 μg, CST, USA) or PIMT antibody (2 μg, Abcam, USA) or Ep300 (1μg, SCBT, USA) or CBP (2μg. CST, USA) bound to protein A/G beads for 2h at 4 °C. Post incubation beads were washed with RIPA lysis buffer followed by boiling with laemmli Buffer. The enriched samples were separated and probed with defined antibodies. According to the manufacturer's instructions, densitometry analysis was performed using freely available Image Studio Lite image processing software (Licor Biosciences) and presented as a target band signal intensity ratio to corresponding input samples.

#### Chromatin immunoprecipitation

Experimental Liver samples were fixed with 1% formaldehyde for 20 min followed by 0.125 M glycine to arrest the cross-linking. The tissues were disaggregated with a Tissue tearor (BioSpec Products, Bartlesville, OK), and chromatin was isolated by adding lysis buffer,^13^ followed by disruption with a Dounce homogenizer. Lysates were sonicated, and the DNA sheared to an average length of 300–500 bp. Genomic DNA (input) was prepared by treating aliquots of chromatin with RNase, proteinase K, and heat for de-crosslinking, followed by ethanol precipitation. Pellets were re-suspended, and the resulting DNA was quantified with a Nanodrop spectrophotometer. Chromatin regions of interest were enriched using an antibody against PIMT (Abcam, USA). Complexes were washed, eluted from the beads with SDS buffer, and subjected to RNase, proteinase-K treatment, and heat for de-crosslinking at 65°C. ChIP DNA was purified by phenol-chloroform extraction and ethanol precipitation. qPCR reactions were carried out in triplicate on specific genomic regions using power SYBR Green mix (Thermo scientific). The resulting signals were normalized to the input DNA. Primer sequences are submitted in key resources table.

#### Polysome profile

Small pieces of the liver (50–100 mg) were homogenized on ice in 5 volumes of polysome buffer (25 mM Tris (HCl), pH 7.4, 10 mM MgCl2, 25 mM NaCl, 0.05% Triton X-100, 0.14M sucrose) containing 100 μg/mL heparin.Nuclei and mitochondria were pelleted by microcentrifugation at 4°C for 10 min. The resulting cytosol preparation was diluted with an equal volume of polysome buffer. 300μL of precleared lysate was fractionated using sucrose gradient (10–50% sucrose) at 35,000 r.p.m. 3 hours at 4 °C using Beckman Coulter SW41 rotor. Polysomal lysis buffer consisted of 20 mM Tris-HCL, 100 mM KCL, 5mM MgCl2, 1%Triton, and sodium deoxycholate 0.25 g. RNA was isolated using Trizol.

#### Real-time PCR

Total RNA was isolated using Tri reagent (Sigma). For qPCR, reverse transcription was performed as described earlier.^13^ The mRNA expression was normalized to reference genes (as mentioned in Figure legends), with the values for control arbitrarily set to 1. Primer sequences are submitted in key resources table.

#### *In vitro*, cellular, and *in vivo* phosphorylation of PIMT

*In vitro* kinase reactions with GST-PIMT fragments were carried out as reported earlier.^13^ In brief, GST-PIMT fragments were incubated with catalytical active PKA as described earlier.^31^ For cellular phosphorylation assay, freshly isolated primary hepatocytes were infected with lentiviral particles expressing pLenti-6.3-PIMT-V5 (wt) or pLenti-6.3-PIMTS565A-V5. Post infections (30h), cells were treated with PKA modulators. Cells were lysed in RIPA lysis buffer.^32^ PKA substrates were immunoprecipitated using Anti-PKA substrate (Cell signaling, CST) bound to protein A/G magnetic beads (Genescript, NJ USA). After 2h incubation at 4°C, the beads were washed three times with lysis buffer and subsequently were reconstituted in Lamelli lysis buffer and separated on 4–12% SDS-PAGE. The separated proteins were transferred to the PVDF membrane and probed by Anti-V5 (Invitrogen, 1:10,000) followed by Anti- GCN5 (1:1000, SCBT). Nonspecific Anti-rabbit IgG (Santacruz Biotechnology Inc., Santacruz, CA, USA) was used as the negative control. For *in vivo* phosphorylation, fasting liver lysates were incubated with PKA substrate antibody for 2h, resolved on 4–12% SDS-PAGE, and probed with anti-PIMT antibody (1:1000)

### Quantification and statistical analysis

Values were expressed as mean ± S.D. For comparison between 2 groups, the unpaired Student's *t-*test was used. One-way ANOVA followed by Bonferroni's post hoc analysis or Dunnett's post hoc analysis compared more than 2 groups. *p*<0.05 was considered significant.

## Data Availability

• All immunoblot analyses were performed with Image Studio Lite as indicated in the STAR Methods sections. No new code is generated in this study. • All data generated or analyzed during this study are included in the present article. • Any additional information required to reanalyze the data reported in this paper is available from the lead contact upon reasonable request.
