# Supplementary material for: PIMT regulates hepatic gluconeogenesis in mice
Source: iScience. 2023 Feb 2;26(3):106120. doi: 10.1016/j.isci.2023.106120 (PMC9972567; doi:10.1016/j.isci.2023.106120)
Supplement: Document S1. Figures S1–S11 [file mmc1.pdf]

iScience, Volume *26*

Supplemental information

PIMT regulates hepatic gluconeogenesis in mice

Bandish Kapadia, Soma Behera, Sireesh T. Kumar, Tapan Shah, Rebecca Kristina Edwin, Phanithi Prakash Babu, Partha Chakrabarti, Kishore V.L. Parsa, and Parimal Misra

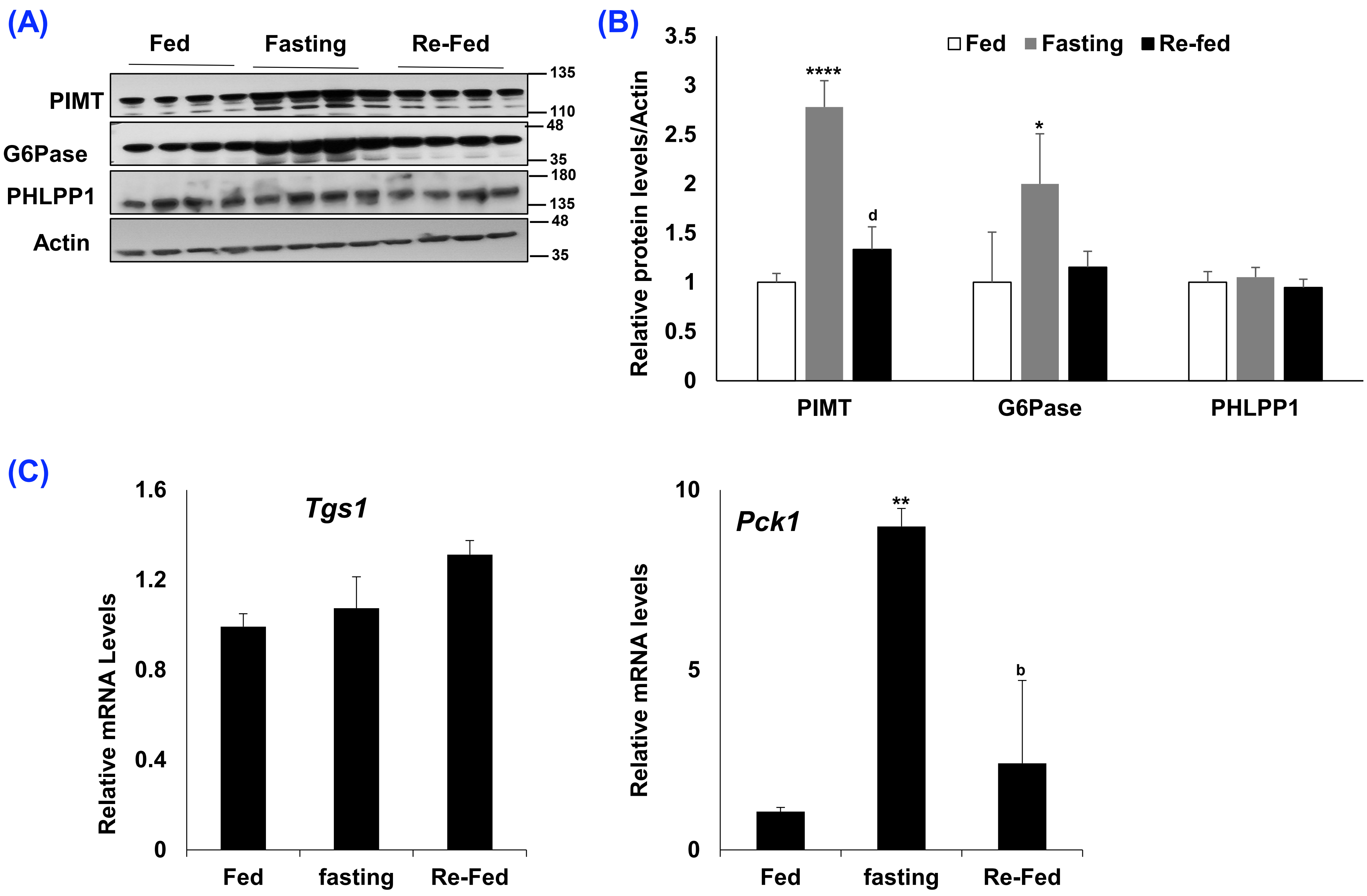

Kapadia et al Supp Fig 1

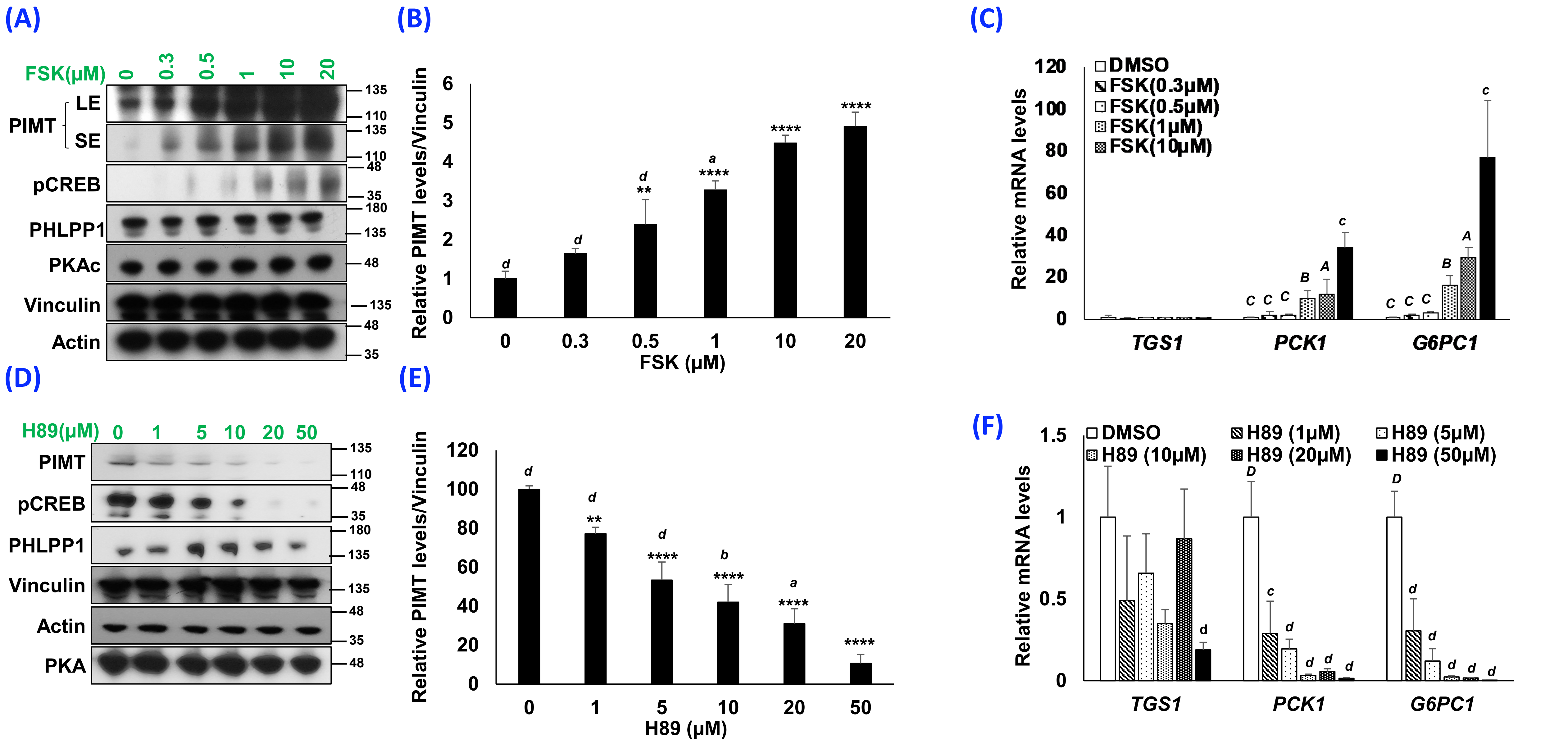

Kapadia et al Supp Fig 2

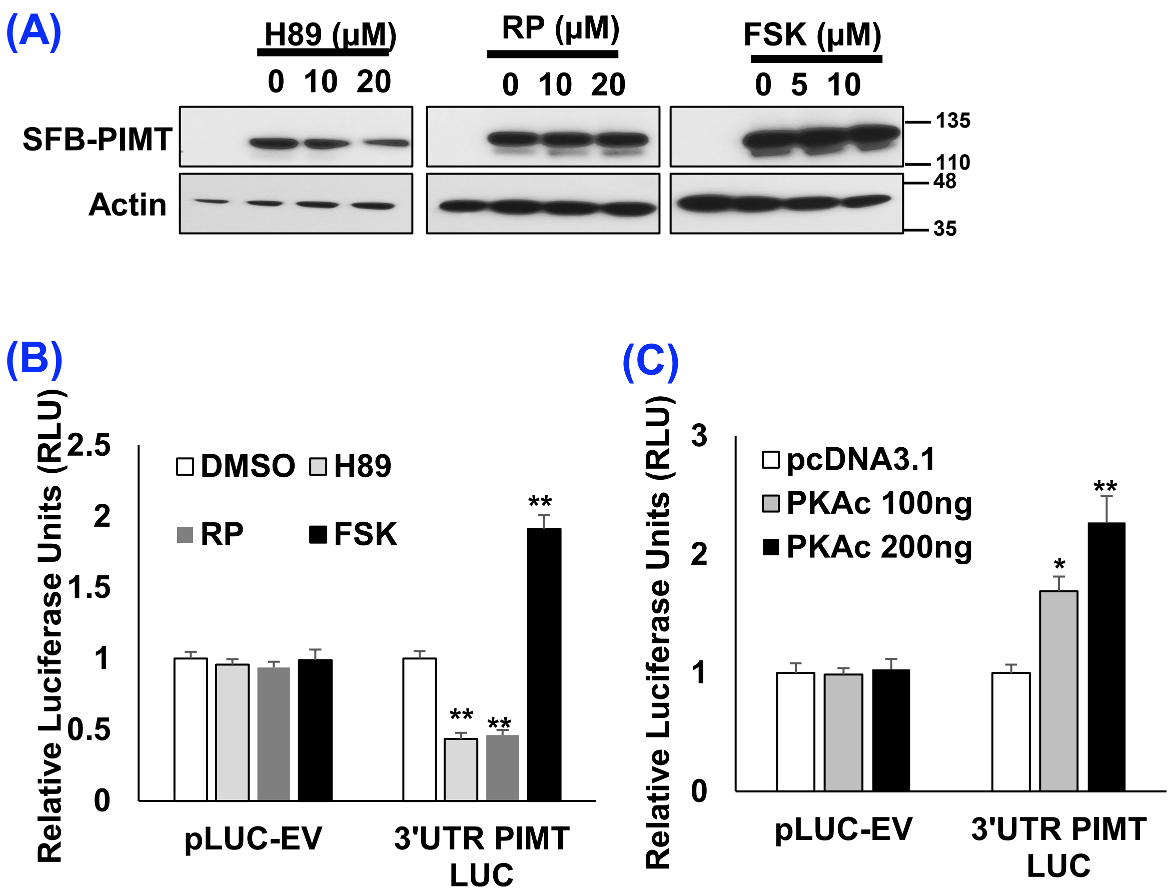

Kapadia et al Supp Fig 3

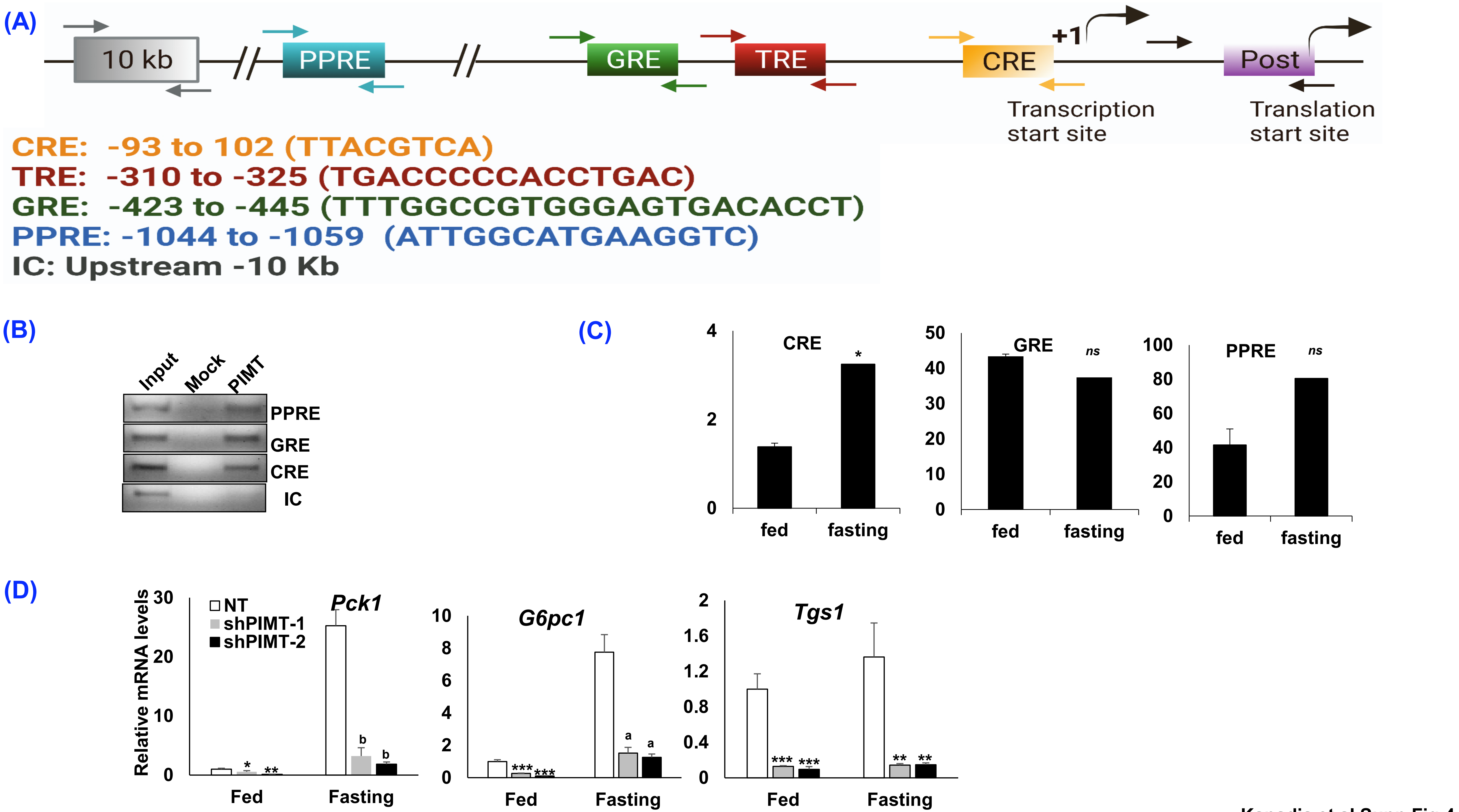

Kapadia et al Supp Fig 4

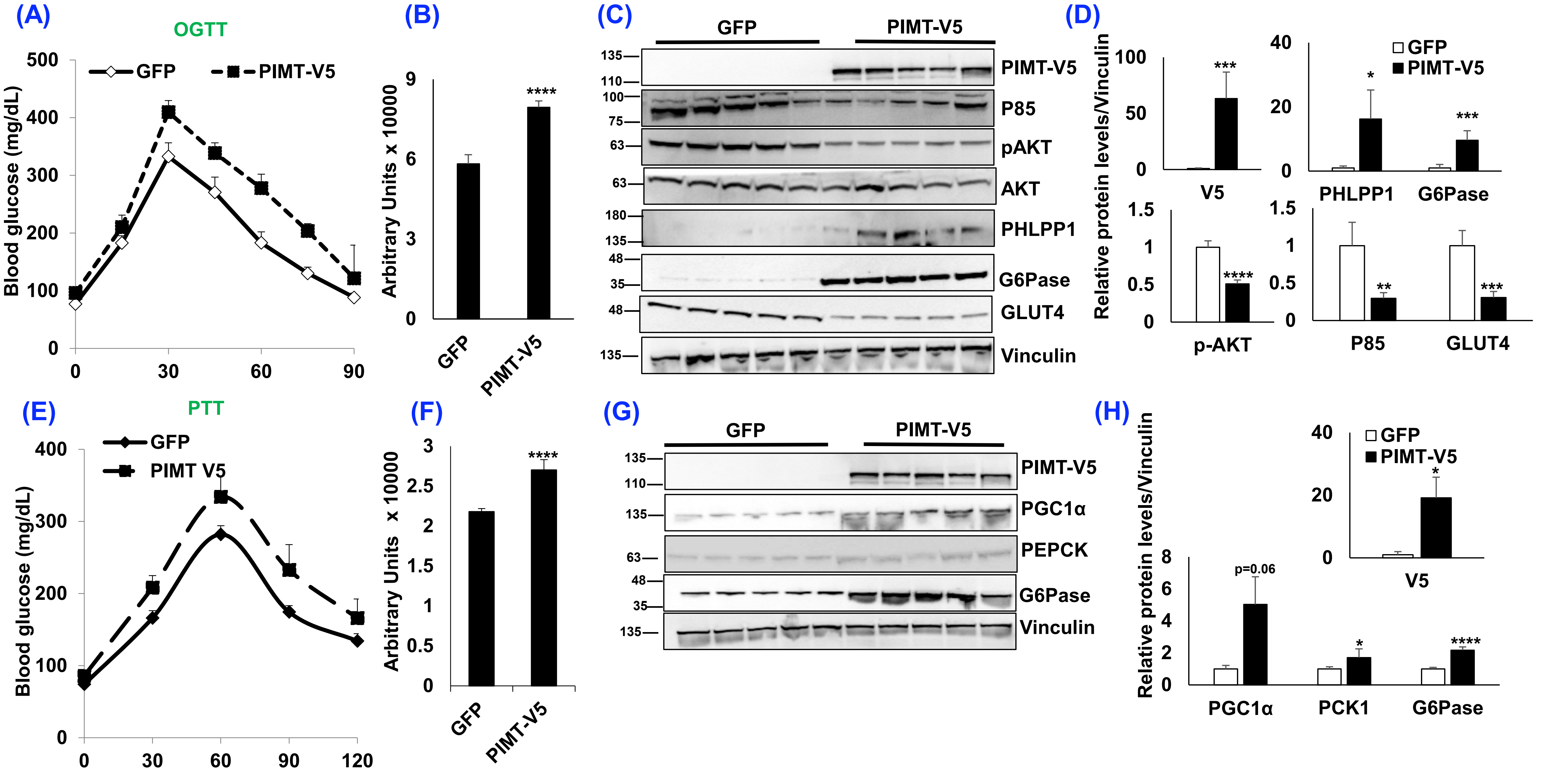

Kapadia et al Supp Fig 5

(A)

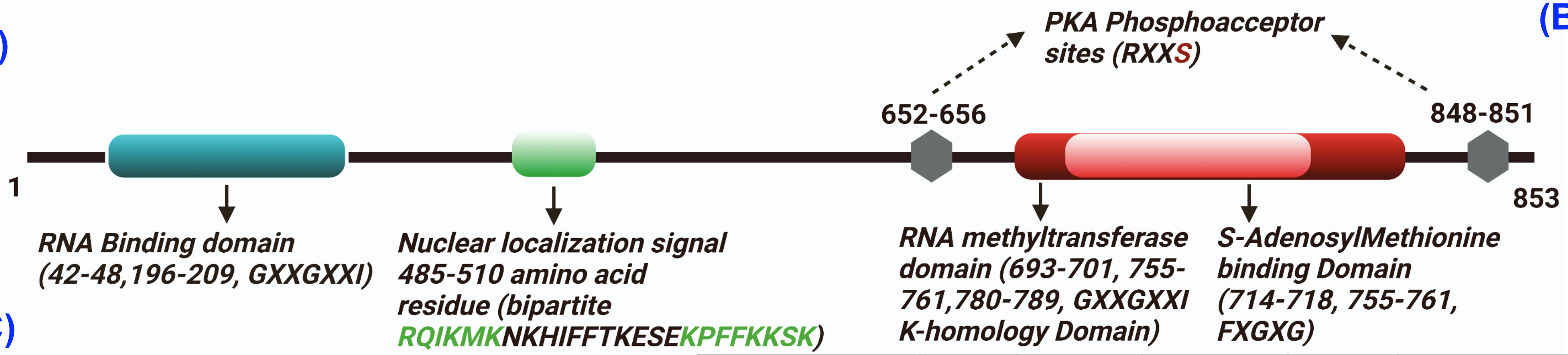

(B) Established PKA substrate alignment

GCN5	QFRQRSQSEDVATYKV
B-RAF	RERKSSSSSEDRNRMKT
PIMT(S656)	RYRLFSRFDDG-IKLDKE
PIMT(S851)	IRRPASET

(C) PKA phosphoacceptor site: 652-656

Homo Sapiens	RYRLFSRFDDG-IKLDKE
Pan troglodytes	RYRLFSRFDDG-IKLDKE
Macaca mulatta	RYRLFSRFDDG-IKLDRE
Canis lupus	RYRLFSRFDDG-IKLDRE
Bos Taurus	RYRLFSRFDDG-IKLDRE
Mus musculus	RYRLFSRFDDG-IKLDRE
Rattus Norvegicus	RYRLFSRFDDG-IKLDRE
Gallus gallus	RYRLFSRFDEG-IKLDRE
Anopheles gambiae	YWKRFSLFSLFDAG-IKL
Danio rerio	RYRLFSRFDEG-IKLDHE
S. cerevisiae	RRRLFSKIDSASIYMTDE

(D) PKA phosphoacceptor site: 848-851

Homo sapiens	TITAYFGDLIRRPASET
Pan troglodytes	TITAYFGDLIRRPASET
Macaca mulatta	TITAYFGDLIRRPASET
Canis lupus familiaris	TITAYFGDLIRRSAPES
Bos taurus	TITAYFGDLIRRSTSES
Mus musculus	TITAYFGDLIRRPALLKTSTSEA
Rattus norvegicus	TITAYFGDLIRRPALRSAEA
Gallus gallus	TITAYFGDLIRPDLS

(E)

Species	Motif Name	Score	Percentile	Site	Sequence	Surface Access Value	DISPHOS	NetPhos Score	NetPhos Kinase
Homo Sapiens	Protein Kinase A	0.705	0.11838	Ser656	aqryrlfsrfddgik	0.6211	YES	0.996	0.67
		NA	NA	Ser851	gdlirrpaset**	NA	YES	0.986	0.67
Pan troglodytes		0.705	0.11838	Ser658	aqryrlfsrfddgik	0.6211	YES	0.996	0.67
		NA	NA	Ser853	dlirrpaset***	NA	YES	0.986	0.67
Macaca mulatta		0.705	0.11838	Ser658	aqryrlfsrfddgik	0.6211	YES	0.996	0.67
		NA	NA	Ser853	rrpaset**	NA	YES	0.986	0.67
Canis lupus		0.705	0.11838	Ser655	aqryrlfsrfddgik	0.6211	YES	0.996	0.67
		NA	NA	Ser849	lirrsapes	NA	YES	0.989	0.67
Bos Tauus		0.705	0.11838	Ser656	aqryrlfsrfddgik	0.6211	YES	0.996	0.67
		NA	NA	Thr850	irrstses*	NA	YES	0.997	0.67
Mus musculus		0.705	0.11838	Ser648	aqryrlfsrfddgik	0.6211	YES	0.996	0.67
		NA	NA	Ser848	lktstseae	NA	NO	0.993	0.67
Rattus Norvegicus		0.705	0.11838	Ser650	aqryrlfsrfddgik	0.6211	YES	0.996	0.67
Gallus gallus		0.699	0.11723	Ser697	aqryrlfsrfdegik	0.6211	YES	0.996	0.67
Danio rerio		0.699	0.11723	Ser609	aqryrlfsrfdegik	0.6211	NO	0.996	0.67
S. cerevisiae		0.631	0.6082	Ser61	knrrrlfskidsasi	0.5134	NO	0.998	0.67

Kapadia et al Supp Fig 6

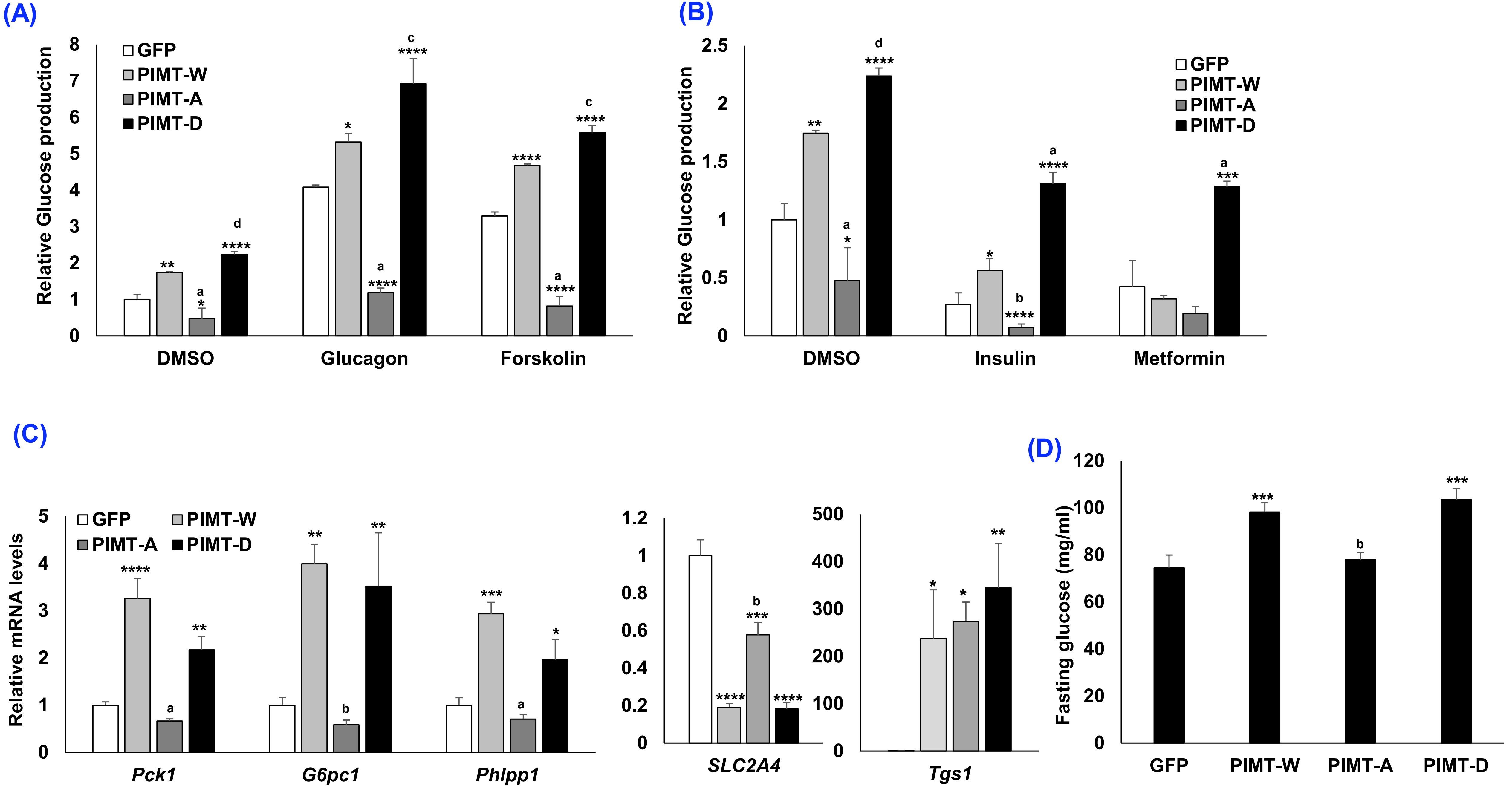

Kapadia et al Supp Fig 7

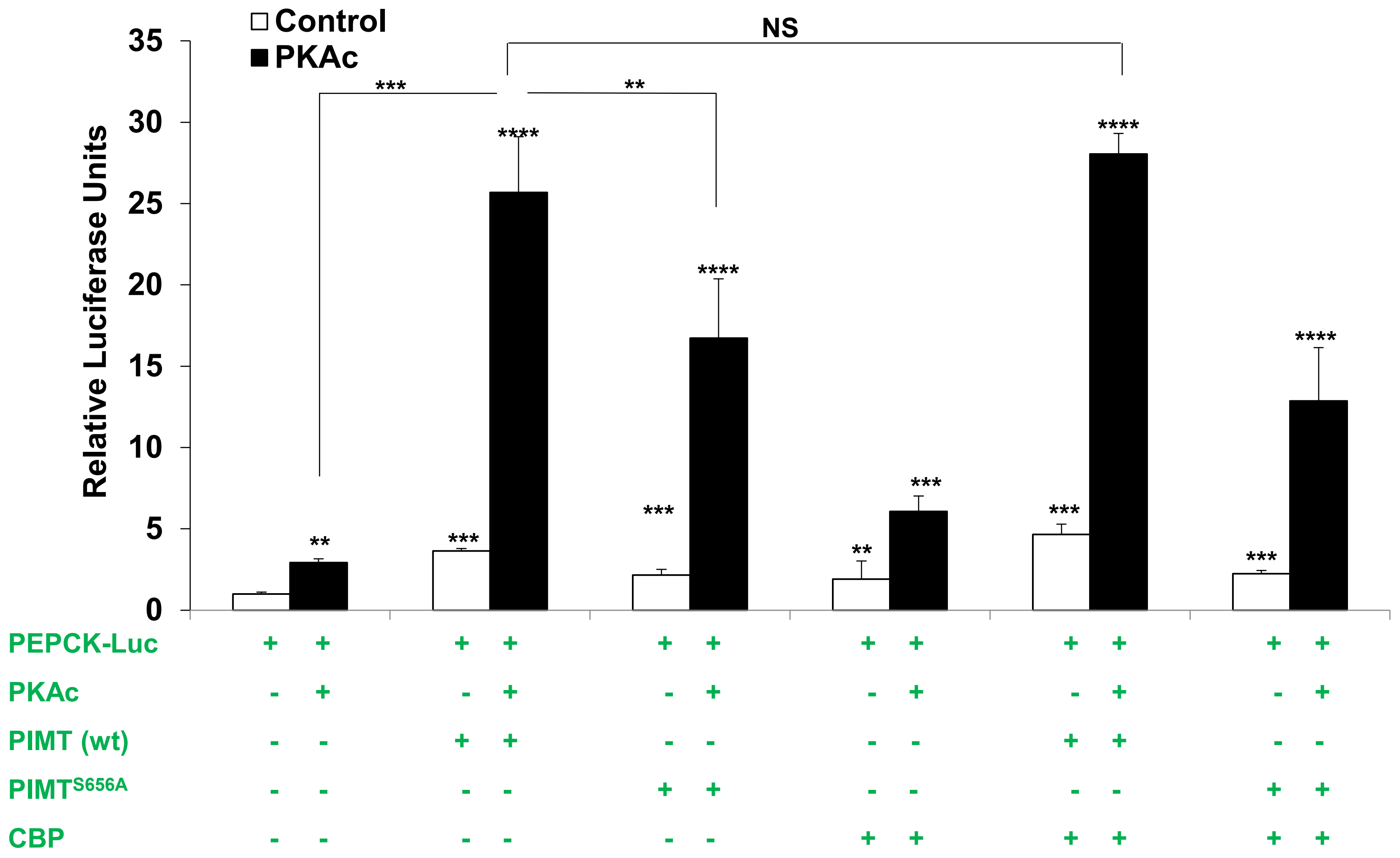

Kapadia et al Supp Fig 8

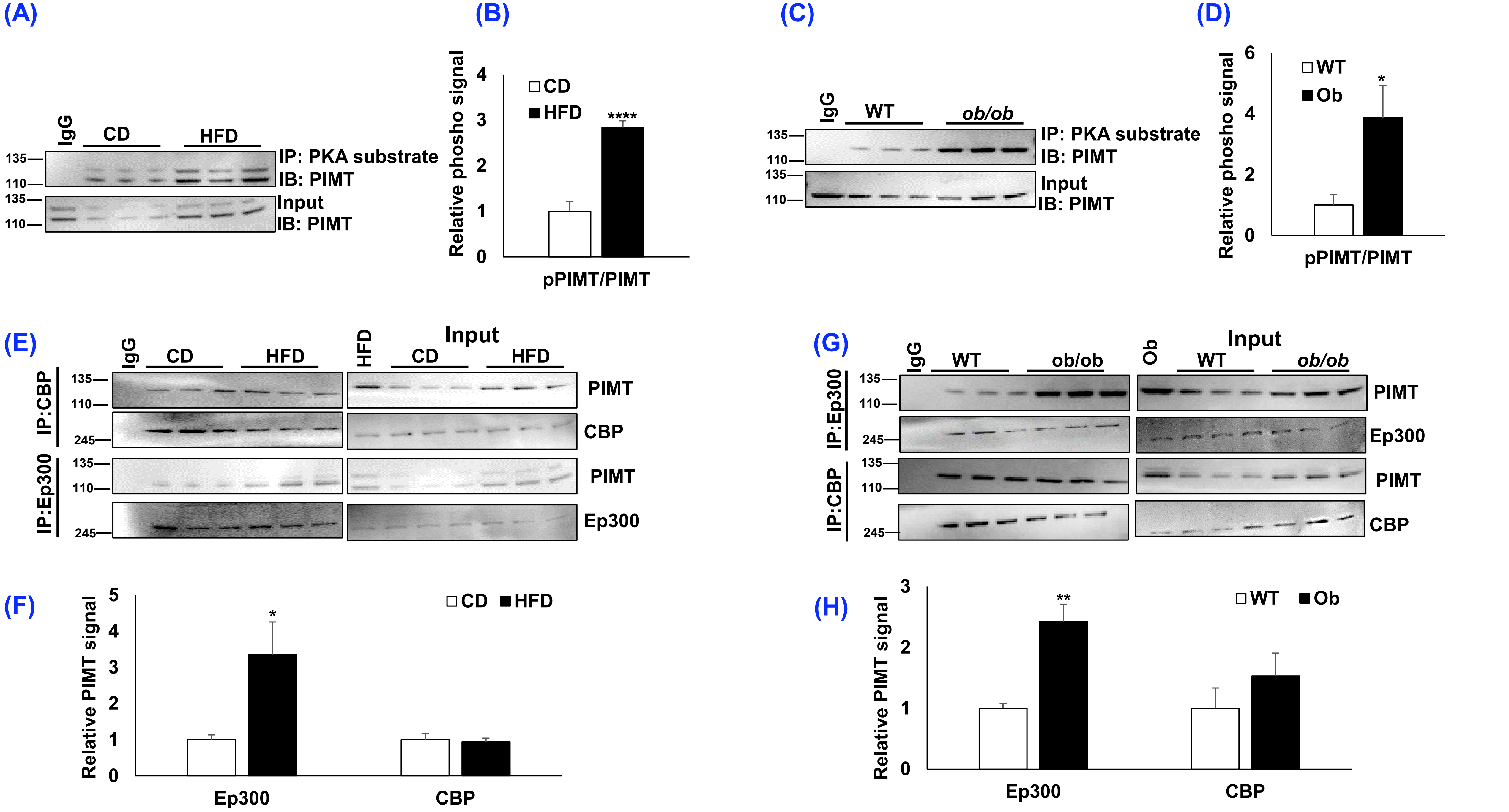

Kapadia et al Supp Fig 9

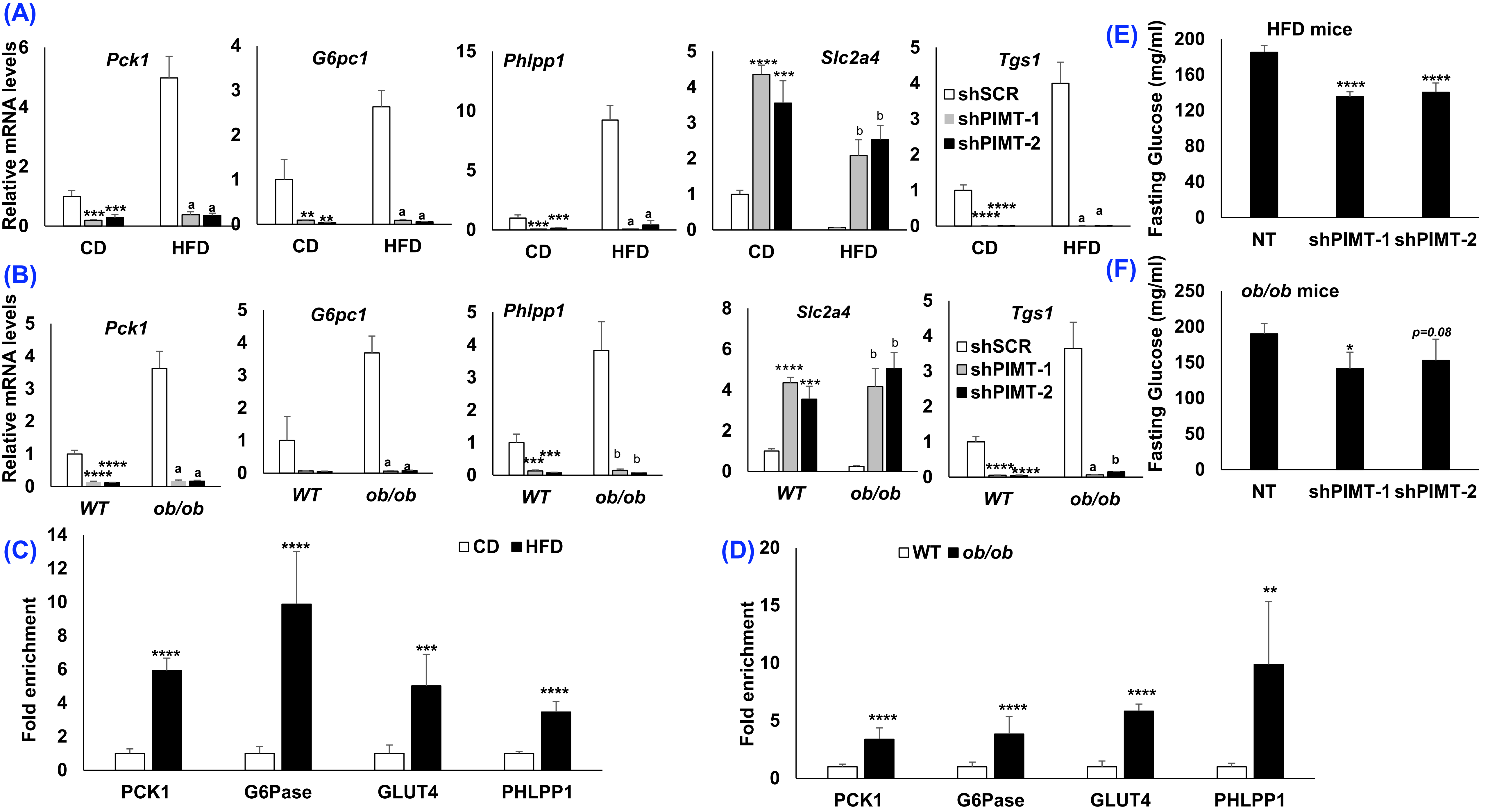

Kapadia et al Supp Fig 10

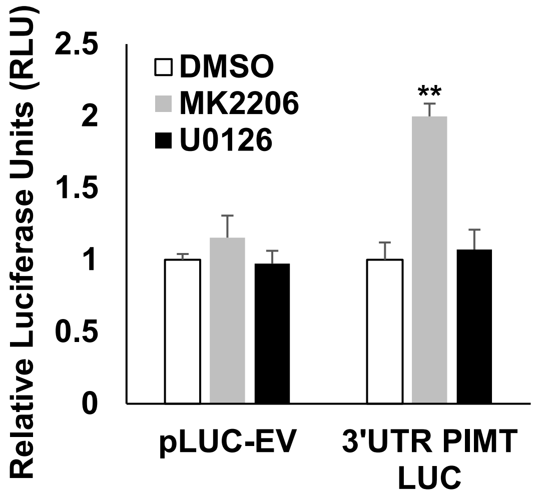

Kapadia et al Supp Fig 11

Legends of the supplementary figures:

Supplemental Figure 1: Nutritional modulation and PIMT expression in mice liver, Related to Figure 1 (A) Immunoblot analysis indicated antibodies in fed, fasted, and fasted/re-fed mice in the liver lysate (n=4). (B) Quantitative densitometry evaluation of the Supp Fig 1A. Values were normalized with the corresponding loading control, actin. Numerical data are expressed as mean ± SD. Statistical analysis was performed using one-way ANOVA followed by Bonferroni's post hoc test. *$p<0.05$, ****$p<0.001$ compared to fed mice, $^{d}p<0.001$ compared to fasted mice. (C) qPCR analysis of the indicated genes in the liver lysate of W and *ob/ob* mice. Values were normalized using 18S as a reference gene and expressed as mean ± SD. Statistical analysis was performed using one-way ANOVA followed by Bonferroni's post hoc test. *$p<0.05$, ****$p<0.001$ compared to fed mice, $^{b}p<0.01$ compared to fasted mice.

Supplemental Figure 2: Hepatic PKA signaling augments PIMT protein levels, related to Figure 2 (A) HepG2 cells were treated with an increasing concentration of Forskolin (FSK). 8h post-treatment, cells were lysed, and immunoblots assessed the protein levels of PIMT (SE: Short exposure, LE: Long exposure). (B) Densitometry quantification of protein PIMT from Supp Fig 2A. Values were normalized with the corresponding loading control, Vinculin. Data are representative of 3 independent experiments and expressed as mean ± SD. Statistical analysis was performed using one-way ANOVA followed by Bonferroni's post hoc test. **$p<0.01$, ****$p<0.001$ compared to control transfected cells,$^{a}p<0.05$, $^{d}p<0.001$ compared to 20µM FSK treated cells. (C) qPCR analysis of the indicated genes upon FSK treatment in HepG2 cells. Values were normalized using 18S as a reference gene. Data are representative of 3 independent experiments and expressed as mean ± SD. Statistical analysis was performed using one-way ANOVA followed by Bonferroni's post hoc test. $^{c}p<0.01$ compared to DMSO treated cells, $^{A}p<0.05$, $^{B}p<0.01$, $^{C}p<0.005$ compared to FSK 20µM treated cells. (D) HepG2 cells were treated with an increasing concentration of H89. Post 8h of treatment, cells were lysed, and immunoblots assessed the protein levels of PIMT. (E) Densitometry quantification of protein PIMT from Supp Fig 2D. Values were normalized with the corresponding loading control, Vinculin. Data are representative of 3 independent experiments and expressed as mean ± SD. Statistical analysis was performed using one-way ANOVA followed by Dunnett's post hoc test. **$p<0.01$, ****$p<0.001$ compared to DMSO treated cells, $^{a}p<0.05$, $^{b}p<0.01$, $^{d}p<0.001$ compared to 50µM H89 treated cells. (F) qPCR analysis of the indicated genes upon H89 treatment in HepG2 cells for 8h. Values were normalized using 18S as a reference gene and expressed as mean ± SD. Statistical analysis was performed using one-way ANOVA followed by Bonferroni's post hoc test. $^{c}p<0.005$, $^{d}p<0.001$ compared to DMSO treated cells,$^{D}p<0.001$ compared to 50µM H89 treated cells.

Supplemental Figure 3: Molecular underpinning of PKA regulated PIMT expression, related to Figure 2 (A) HepG2 cells were transfected with SFB-PIMT encoding construct. Post 36h of transfection, cells were treated with an increasing concentration of the indicated compounds. Post-treatment, cells were lysed, and the levels of ectopic PIMT were determined using anti-Flag antibodies by immunoblots. (B) HepG2 cells were transfected with the pLenti-PIMT 3'UTR-Luc reporter construct. Post 36h of transfection, cells were treated with the increasing concentration of the indicated compounds (H89-10µM, RP-10µM, FSK 5µM). Post-treatment, cells were lysed, and luciferase signals were quantified. Renilla luciferase signals were used as

an internal control. The values were normalized with corresponding Renilla luciferase activity and expressed relative to DMSO treated cells (column 1) set to 1. pLenti-Empty-Luc reporter construct was the negative control. Data are representative of 5 independent experiments and expressed as mean ± SD. Statistical analysis was performed using one-way ANOVA followed by Dunnett's post hoc test. **$p<0.01$ compared to DMSO-treated cells. (C) HepG2 cells were transfected with pLenti-PIMT 3'UTR-Luc reporter constructs along with the increasing concentration of PKAc. Post 36h of transfection, cells were lysed, and luciferase signals were quantified. Renilla luciferase signals were used as an internal control. The values were normalized with corresponding Renilla luciferase activity. They were expressed relative to DMSO-treated cells (column 1) set to 1. pLenti-Empty-Luc reporter construct was the negative control. Numerical data are expressed as mean ± SD. Statistical analysis was performed using one-way ANOVA followed by Dunnett's post hoc test. *$p<0.05$, **$p<0.01$ compared to DMSO treated cells.

Supplemental Figure 4: PIMT regulated gluconeogenesis *in vivo*, related to Figure 3 (A) Schematic illustration of mice PEPCK promoter representing different responsive elements. CRE: cAMP-responsive element, TRE: Thyroid Responsive element, GRE: Glucocorticoid responsive element, PPRE: PPAR response element, IC: Internal control (negative control). (B) Chromatin immunoprecipitation was performed using anti-PIMT or nonspecific IgG antibody on liver lysate from fed mice PEPCK promoter. Indicated genes were amplified by PCR and image and separated on agarose gels. (C). Chromatin immunoprecipitation was performed using anti-PIMT or nonspecific IgG antibody on liver lysate from fed or fasted mice PEPCK promoter. Indicated regions of PEPCK promoter were quantified by qPCR. Numerical data are expressed as mean ± SD. Statistical analysis was performed using unpaired Student's t-test (two-tailed) *$p<0.05$ versus the corresponding fed mice. (D) C57BL/6 mice were tail-vein injected with lentivirus expressing shRNA against *Tgs1* (two independent shRNA). shSCR tail-vein injections were used as the internal control. After 7 days of injection, mice fasted for 8h. Post-fasting, mice were euthanized, and expression of indicated genes in the liver was quantified by qPCR. Values were normalized using 18S as a reference gene and expressed as mean ± SD. Statistical analysis was performed using one-way ANOVA followed by Dunnett's post hoc test. *$p<0.05$, **$p<0.01$, ***$p<0.005$ compared to control fed mice, a$p<0.001$, b$p<0.005$ compared to control fasting mice.

Supplemental Figure 5: Overexpresion of PIMT enhances hepatic glucose production, related to Figure 3 (A)Oral Glucose tolerance test in C57BL/6J mice tail vein injected with lentivirus expressing PIMT (W) or GFP in the liver (n=5). (B) The area under the curve for Supp Fig 5A. Numerical data are expressed as mean ± SD.Statistical analysis was performed using unpaired Student's t-test (two-tailed), ****$p<0.001$ versus the GFP-injected mice. (C) Immunoblot analysis of the liver lysates using the indicated antibodies. (D) Densitometric quantification of Supp Fig 5C. Values were normalized with the corresponding loading control, Vinculin. Numerical data are expressed as mean ± SD. Statistical analysis was performed using unpaired Student's *t*-test (two-tailed), ****$p<0.001$ versus the GFP-injected mice. (E) Pyruvate tolerance test in C57BL/6J mice tail vein injected with lentivirus expressing PIMT (W) or GFP in the liver. (F) The area under the curve for Supp Fig 5E. Numerical data are expressed as mean ± SD. Statistical analysis was performed using unpaired Student's *t*-test (two-tailed), ****$p<0.001$ versus the GFP-injected mice. (G) Immunoblot analysis of the liver

lysates using the indicated antibodies. (H) Densitometric quantification of Supp Fig 5G. Values were normalized with the corresponding loading control, Vinculin. Numerical data are expressed as mean ± SD. Statistical analysis was performed using unpaired Student's *t*-test (two-tailed), $^{**}p<0.01$, $^{**}p<0.01$, $^{***}p<0.005$, $^{****}p<0.001$ compared to GFP-infected mice. ependent experiments unless specified. Numerical data are expressed as mean ± SD.

Supplemental Figure 6: PIMT as a potential PKA substrate, related to Figure 4
(A) Schematic diagram of PIMT protein domain. PIMT contains an RNA binding domain, an S-adenosyl methionine binding domain overlapping with the RNA Methyltransferase domain. The location of RxxS sites (target site of PKA) is also indicated. (B) Alignment of established PKA substrates with potential PIMT PKA phosphoacceptor site. (C) PIMTSer^{656} is evolutionary conserved across different species. (D) PIMTSer^{851} is conserved only in higher vertebrates. (E) Prediction of PIMT as a substrate of PKA using ScanSite 3.0, DISPHOS and NetPhos 2.0, and NetphosK 1.0.

Supplemental Figure 7: PKA-mediated PIMT phosphorylation is a hepatic hyperglycemic driver in vivo, related to Figure 5 (A, B) Freshly isolated primary hepatocytes from independent four female mice infected with lentiviruses expression of either GFP or PIMT (W and mutants). Post 48h of infections, cells were washed and cultured in glucose production media along with indicated treatments (Glucagon: 100nM, Forskolin: 10uM, Insulin:10nM, Metformin 1µM). Six hours post-treatment, secreted glucose was quantified from the media. Numerical data are expressed as mean ± SD. Statistical analysis was performed using one-way ANOVA followed by Bonferroni's post hoc test. $^{*}p<0.05$, $^{**}p<0.01$, $^{***}p<0.005$, $^{****}p<0.001$ compared to GFP-infected cells, $^{d}p<0.05$, $^{c}p<0.01$, b$p<0.005$, $^{a}p<0.001$ compared to PIMT-W-infected cells. (C) C57BL/6 mice were tail-vein injected with lentivirus expressing either GFP or PIMT (W and mutants). Post 7 days of injection, mice were euthanized, and expression of indicated genes in the liver was quantified by qPCR. Values were normalized using 18S as a reference gene and are expressed as mean ± SD. Statistical analysis was performed using one-way ANOVA followed by Bonferroni's post hoc test. $^{*}p<0.05$, $^{**}p<0.01$, $^{***}p<0.005$, $^{****}p<0.001$ compared to GFP-infected cells, b$p<0.005$, $^{a}p<0.001$ compared to PIMT-W-infected cells (D) C57BL/6 mice were tail-vein injected with lentivirus expressing either GFP or PIMT (W and mutants) (n=4). Post 7 days of injection, glucose was quantified from the tail vein. Numerical data are expressed as mean ± SD. Statistical analysis was performed using one-way ANOVA followed by Bonferroni's post hoc test. $^{***}p<0.005$, compared to GFP-infected cells, b$p<0.005$ compared to PIMT-W-infected cells. PIMT-W: PIMT wild type, PIMT-A: PIMT S656A mutant, and PIMT-D: PIMT S656D mutant.

Supplemental Figure 8: PKA regulates PIMT-Ep300, but not PIMT-CBP, transactivation activity through phosphorylation, related Figure 6
HepG2 cells were transfected with pGL3-PEPCK-Luc promoter along with CBP, and PIMT (W and mutants) in the presence or absence of PKAc. Post 36h of transfection, cells were lysed, and luciferase signals were quantified. Renilla luciferase signals were used as an internal control. The values were normalized with corresponding Renilla luciferase activity and expressed relative to PEPCK-Luc (unphosphorylated) (column 1), set to 1. Numerical data are expressed as mean ± SD. Statistical analysis was

performed using one-way ANOVA followed by Bonferroni's post hoc test. **p<0.01, ***p<0.005, ****p<0.001 NS non-significant.

Supplemental Figure 9: Obesity enhances PIMT-Ep300 complex formation, related Figure 7 (A, C) Liver lysates from HFD mice (A) or *ob/ob* mice (C) subjected to IP with PKA substrate antibody followed by immunoblots with the defined antibodies. Chow Fed mice (A) and WT mice (C) were used as internal controls (B, D) (n=3). Densitometric quantification of Supplemental Fig 9A and 9C. The phosphorylation signals were normalized with the corresponding input signals. Numerical data are expressed as mean ± SD. Statistical analysis was performed using unpaired Student's t-test (two-tailed) *p<0.05, ****p<0.01, versus the corresponding input signals. (E, G) Liver lysates from HFD mice (E) or ob/ob mice (G)were subjected to IP with anti-CBP or anti-Ep300 antibodies followed by immunoblots with the defined antibodies. Chow Fed mice (E) and WT mice (G) were used as internal controls. (F, H) Densitometric quantification of Supplemental Fig 9F and 9G. The interaction signals were normalized with the corresponding enriched protein signals. Numerical data are expressed as mean ± SD. Statistical analysis was performed using unpaired Student's t-test (two-tailed) *p<0.05, **p<0.01, versus the corresponding control mice.

Supplemental Figure 10: Suppression of PIMT ameliorates diabetes, related to Figure 7 (A, B) Diabetic mice models: HFD (A) or *ob/ob* (B) were tail-vein injected with lentivirus expressing shRNA against *Tgs1*(two independent shRNA). shSCR tail-vein injections were used as the internal control. Chow Fed mice (A) and W mice (B) were used as negative controls. Post 7 days of injection, mice were euthanized, and expression of indicated genes in the liver was quantified by qPCR (n=5). Values were normalized using 18S as a reference gene and are expressed as mean ± SD.. Statistical analysis was performed using one-way ANOVA followed by Bonferroni's post hoc test. ***p<0.005, ****p<0.001 compared to corresponding control mice, bp<0.005, ap<0.001 compared to corresponding diabetic mice. (C, D) Chromatin immunoprecipitation was performed using anti-PIMT or nonspecific IgG antibody on liver lysate from HFD (C) or *ob/ob* (D) mice. Chow Fed mice (A) and W mice (C) were used as internal controls. Indicated genes promoter were quantified by qPCR. Numerical data are expressed as mean ± SD. Statistical analysis was performed using unpaired Student’s t-test (two-tailed) **p<0.01, ***p<0.005, ****p<0.001 versus the corresponding control mice. (E, F) Diabetic mice models: HFD (E) or *ob/ob* (F) were tail-vein injected with lentivirus expressing shRNA against *Tgs1* (two independent shRNA). shSCR tail-vein injections were used as the internal control. Post 7 days of injection, fasting glucose was quantified from the tail vein. Numerical data are expressed as mean ± SD. Statistical analysis was performed using one-way ANOVA followed by Dunnett's post hoc test. *p<0.05, ****p<0.001 compared to control fasting mice.

Supplemental Figure 11: Akt activity hampers PIMT 3’ UTR driven translation, related to Figure 7. HepG2 cells were transfected with pLenti-PIMT 3’UTR-Luc reporter construct. Post 36h of transfection, cells were treated with the increasing concentration of the indicated compounds (MK2206-300nM, UO126: 10µM). Post-treatment, cells were lysed, and luciferase signals were quantified. Renilla luciferase signals were used as an internal control. The values were normalized with corresponding Renilla luciferase activity. They expressed relative to DMSO treated cells (column 1) set to 1. pLenti-Empty-Luc reporter construct was the negative control.

Numerical data are expressed as mean ± SD. Statistical analysis was performed using one-way ANOVA followed by Dunnett's post hoc test. $^{**}p<0.01$ compared to DMSO-treated cells.
